# Light-dependent variations in fatty acid profiles and gene expression in Antarctic microalgal cultures

**DOI:** 10.1371/journal.pone.0317044

**Published:** 2025-01-16

**Authors:** Jacqui Stuart, Kirsty F. Smith, Matt Miller, John K. Pearman, Natalie Robinson, Lesley Rhodes, Lucy Thompson, Sarah Challenger, Nicole Parnell, Ken G. Ryan

**Affiliations:** 1 Victoria University of Wellington, Wellington, New Zealand; 2 Cawthron Institute, Nelson, New Zealand; 3 National Institute of Water and Atmospheric Research (NIWA), Wellington, New Zealand; 4 Lincoln University, Lincoln, New Zealand; University of Innsbruck, AUSTRIA

## Abstract

Photosynthetic eukaryotic microalgae are key primary producers in the Antarctic sea ice environment. Anticipated changes in sea ice thickness and snow load due to climate change may cause substantial shifts in available light to these ice-associated organisms. This study used a laboratory-based experiment to investigate how light levels, simulating different sea ice and snow thicknesses, affect fatty acid (FA) composition in two ice associated microalgae species, the pennate diatom *Nitzschia* cf. *biundulata* and the dinoflagellate *Polarella glacialis*. FA profiling and transcriptomic analyses were used to compare the impact of three light levels: High (baseline culturing conditions 90 ± 1 μmol photons m^−2^ s^−1^), mid (10 ± 1 μmol photons m^−2^ s^−1^); and low (1.5 ± 1 μmol photons m^−2^ s^−1^) on each isolate. Both microalgal isolates had altered growth rates and shifts in FA composition under different light conditions. *Nitzschia* cf. *biundulata* exhibited significant changes in specific saturated and monounsaturated FAs, with a notable increase in energy storage-related FAs under conditions emulating thinner ice or reduced snow cover. *Polarella glacialis* significantly increased production of polyunsaturated FAs (PUFAs) in mid light conditions, particularly octadecapentaenoic acid (C18:5N-3), indicating enhanced membrane fluidity and synthesis of longer-chain PUFAs. Notably, C18:5N-3 has been identified as an ichthyotoxic molecule, with fish mortalities associated with other high producing marine taxa. High light levels caused down regulation of photosynthetic genes in *N*. cf. *biundulata* isolates and up-regulation in *P*. *glacialis* isolates. This and the FA composition changes show the variability of acclimation strategies for different taxonomic groups, providing insights into the responses of microalgae to light stress. This variability could impact polar food webs under climate change, particularly through changes in macronutrient availability to higher trophic levels due to species specific acclimation responses. Further research on the broader microalgal community is needed to clarify the extent of these effects.

## Introduction

Photosynthetic eukaryotic microalgae support marine biodiversity and biochemical processes in polar ecosystems. These organisms are incorporated into the sea ice column as it forms. They grow in the liquid phase between ice crystals, and become a key macronutrient source for many organisms during the frozen winter and following spring [1–3]. Ice-associated algal communities can persist through summer and autumn, but their contribution to macronutrients is significantly reduced compared to the dominant pelagic phytoplankton [2, 3]. Though polar microalgae are capable of withstanding extreme natural seasonal variability [4], the increased pressure on polar ecosystems due to the changing climate [5], has begun to influence ice associated microalgae community composition [6].

Microalgal growth is driven by many abiotic factors, such as light, temperature, nutrients, and salinity [7, 8]. Of these, light is the most important factor for biomass growth and accumulation in auto-trophic photosynthetic microalgae [9–11]. Light availability in sea ice habitats is directly influenced by the thickness of sea ice and associated snow cover [4, 12–14]. Snow plays a substantial role in regulating under-ice light intensities due to its high albedo, effectively reducing light penetration through the ice below [4, 13].

Snow cover and depth in Antarctica is coupled with the age and duration of sea ice cover [15]. With climate change predicted to reduce sea ice coverage, duration and thickness around Antarctica [16], there may be less time for snow to accumulate. This could lead to increased light penetration during ice algae growing season, however the extent to which light availability will change remains uncertain. The growing season for ice associated microalgae is currently only a few months, and is likely to become more time restricted in coming years [17].

**Fig 1:**
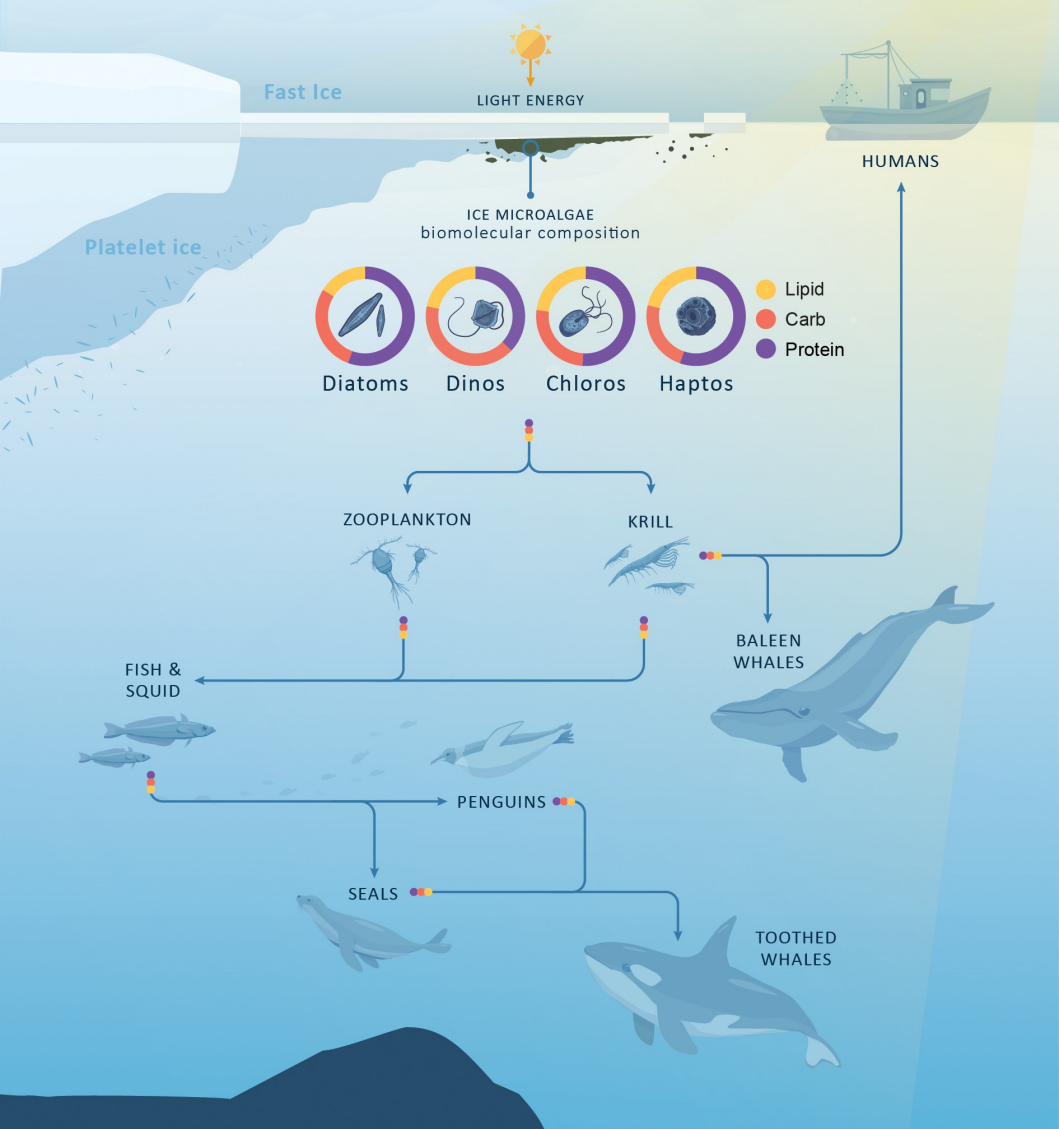
Schematic of biomolecular composition for high-level microalgae taxonomic groups and the flow of these through the Antarctic food web. Microalgae taxonomic groups include diatoms, dinoflagellates (dinos), chlorophytes (chloros) and haptophytes (haptos). Biomolecular composition data adapted from [27]. Though Krill are a component of the zooplankton community, they have been highlighted separately here to show their importance for whales and humans.

Eukaryotic microalgal communities within polar marine ecosystems are the main source of biomolecules in this environment [18]. These molecules include proteins, carbohydrates, and lipids, which are all essential for the functioning of the Antarctic marine food web [3, 19]. Within microalgal cells, the allocation of photosynthates to the synthesis of various biomolecules is largely influenced by environmental conditions [19]. This means that changes in environmental conditions can impact the quantity and composition of proteins, carbohydrates, and lipids available to primary grazers such as krill and zooplankton [3, 20, 21] (Fig 1).

Environmental variability, both within expected ranges and climate change enhanced, can induce shifts in the composition of eukaryotic microalgal communities [4, 6]. Within the eukaryotic microalgal communities, the relative proportion of different high-level taxonomic groups (e.g. class level) can be the largest driver of biomolecular variation in an ecosystem. Fatty acids (FAs) are the densest form of energy of these biomolecules [22]. Different types of microalgae exhibit distinct FA profiles [23, 24]. Pennate diatoms (Bacillariophyceae) for example, have higher levels of certain FAs compared to other taxa such as polar centric diatoms, chlorophytes or haptophytes [25]. Antarctic fast ice and the sub-ice platelet layer ecosystems are dominated by pennate species [26].

Most experimental research into shifts in FA composition associated with light levels for Antarctic microalgae has focused on mixed diatom species [28, 29], while a far smaller number include haptophytes [30] and chlorophytes [31]. Dinoflagellates have not been studied using manipulative experiments to date. Though diatoms do contribute substantially to the ice microalgal community in many cases, dinoflagellates can make up a larger proportion of the eukaryotic microalgal community in the mid and upper extents of sea ice [6, 32]. Within sea and platelet ice environments dinoflagellates consistently contribute more biomass than both chlorophytes and haptophytes, but not diatoms [6, 32].

As FAs are important macronutrients in the polar marine environment, we undertook a laboratory-based experiment to assess the impact of shifting light levels on FA production in two key microalgal groups (pennate diatoms and dinoflagellates) from sea ice eukaryotic microalgal communities. Both pennate diatoms and dinoflagellates have shown substantial shifts in relative abundance within eukaryotic microalgal communities of sea ice and associated under shifting ice regimes in Antarctica [6]. With this experiment, we aimed to investigate: 1) if light conditions that reflect different sea ice thickness change FA composition in two microalgal isolates from key taxonomic groups; 2) how these changes are expressed in FA and photosynthetic biosynthetic gene pathways; and 3) what this may imply for sea ice eukaryotic microalgal community composition changes and their impact on available FAs in the food web. Understanding and quantifying the biomolecular composition of microalgal species from key taxonomic groups will contribute to identifying how environmental conditions impact the synthesis of microalgae biomolecules. These in turn can be applied to analyse observations in broader ice associated eukaryotic microalgal community biomolecular characterisations.

## Materials and methods

### Microalgae isolates and growing conditions

Two microalgal strains were used, one pennate diatom *Nitzschia* cf. *biundulata* (CAWB171; S1A Fig in S1 File) and one dinoflagellate *Polarella glacialis* (CAWD459; S1B Fig in S1 File). Both were isolated from samples collected in Cape Evans, McMurdo Sound, Antarctica (-77.636, 166.377) in November 2021 (*N*. cf. *biundulata*) and November 2022 (*P*. *glacialis*). The dinoflagellate *P*. *glacialis* was isolated from brine water samples. A hole was drilled in the sea ice to 0.5 m depth, then left for 30 minutes to allow brine to drain into the hole. The pennate diatom *N*. cf. *biundulata* was isolated from melted ice scraped from the bottom of sea ice cores. Samples were collected and transported under the permit 2022080076 granted by the New Zealand Ministry for Primary Industries (Manatū Ahu Matua). Cultures were isolated and maintained at the Cawthron Institute Culture Collection of Microalgae (CICCM; http://cultures.cawthron.org.nz/ciccm/). Both cultures were maintained in f/2 media [33] at 35.3 ppt salinity, under 90 ± 1 μmol photons m^−2^ s^−1^ irradiance (12:12h Light:Dark photoperiod) at 4 ± 1°C. Small sub-unit rDNA sequences for each isolate used in this experiment were submitted to GenBank under accession numbers PP928079 (*N*. *cf*. *biundulata*) and PP922274 (*P*. *glacialis*). These isolates were selected due to their prevalence in field samples.

### Experiment design and setup

General culturing conditions described above served as the high light treatment. These growing conditions may seem high for microalgae from sea ice, however, they can experience similar or even greater irradiance levels during late spring melt or in future thinning ice scenarios [4]. This variability underscores the relevance of our chosen experimental conditions for assessing microalgal responses. Two additional light treatments were also used, mid light (10 ± 1 μmol photons m^−2^ s^−1^) and low light (1.5 ± 1 μmol photons m^−2^ s^−1^) (S2 Table in S1 File). These light levels are reflective of natural light intensities sea ice algae can experience under consolidated fast ice, dependant on ice thickness and snow cover. Seed cultures for each isolate were grown in general culturing conditions, with media refreshed every two weeks. Once enough biomass was accumulated, a total of fifteen cultures were inoculated with 8,000–10,000 cells/mL in 400 mL of f/2 media for both *N*. *cf*. *biundulata* and *P*. *glacialis* isolates. Five replicate cultures of each isolate were then acclimated to their respective light treatments for seven days before the experiment began. Cell counts were completed every two days to assess growth rates, with each replicate harvested once they reached exponential growth phase. The low light treatment for *P*. *glacialis* was harvested after 56 days as growth had plateaued.

### FA extraction and identification

At the point of harvest 280–300 mL of *P*. *glacialis* and *Nitzschia* sp. replicates were centrifuged (3000 x *g* for 10 minutes) in 50 mL falcon tubes (Corning CentriStar, China). Pellets were stored at –20°C until FAs were extracted. Each pellet was freeze-dried (*Martin*Martin *Christ*, *Germany)* and weighed. Algal pellets (3–30 mg; S3 Table in S1 File) were dried and processed for FA methyl esters (FAMEs) using gas chromatography (GC) analysis as defined in [34]. FA composition was established as defined in [35], with the relative response factor (RRF) of each peak determined from a commercial standard of equal weight [35].

In our results, total proportional FAs and the contributions of polyunsaturated (PUFA), monounsaturated (MUFA), and saturated fatty acids (SFA) are assessed. SFAs have no double bonds between carbon atoms, MUFAs have one double bond, and PUFAs contain two or more double bonds, each playing distinct biological roles. The Omega nomenclature are used to express FAs in the results section, figures and discussion.

### RNA extraction

To prepare samples for RNA extractions 100 mL of culture from each replicate (n = 15) was vacuum filtered (S2 Table in S1 File) using 0.45 μm PVDF membrane filters (Merck, Ireland). Samples were snap frozen in liquid nitrogen and immediately stored at -80°C until extraction. Extraction of RNA was complete in sterile conditions using the RNeasy Plant Mini Kit (Qiagen, Germany) per manufacturer’s instructions with the addition of an initial homogenisation step. Samples were homogenised using ZR BashingBead Lysis Tubes (0.1 mm beads; Zymo Research, USA) with Cell Lysis Buffer and the addition of β-mercaptoethanol, as directed in the kit protocol in a 1600 MiniG Spex SamplePrep machine (New Jersey, United States) for 2 mins at 1500 rpm. DNase treatments were then undertaken with a TURBO DNA-*free* Kit (ThermoFisher Scientific), following the manufacturer’s instructions to remove any remaining DNA contamination. Immediately post DNase treatment, RNA quantification and quality checks were undertaken. The concentration was assessed using a NP80 nanophotometer (Implen, Munich) and quality was visualised on 1.5% agarose gel stained with RedSafe Nucleic Acid Staining Solution (iNtRON Biotechnology, Korea). Samples were then dried and sent to GENEWIZ (AZENTA Life Sciences, Suzhou, China) for subsequent RNA-seq library preparation. Sequencing was completed by GENEWIZ using the Illumina™ NovaSeq 6000 Sequencing System with 150 pair end reads, at a minimum of 20M PE raw reads per sample.

### Bioinformatic analysis

Both DNA isolates used in this experiment went through the following bioinformatic and statistical analysis steps separately. Any difference in processing of isolates is highlighted. Assembly of a *de novo* transcriptome was undertaken via the Oyster River Protocol (ORP) [36] from 9 of the replicates for each isolate. Three random samples from each treatment were selected. The ORP removes sequencing adaptors (*Trimmomatic* [37]) and filters for quality (Phred <2) before correcting reads (*RCorrector* [38]). Three unique assemblies were then created using the following assemblers: *TransAbyss* v2.0.1 [39, 40] and two *SPAdes* v3.15.2 [41] assemblies using a kmer length of 55 and 75. A single high quality assembly was then produced by merging all assemblies (*OrthoFuse* [36]). This was then assessed using Benchmarking sets of Universal Single-Copy Orthologs (BUSCO v5.4.4 [42]) with the Odb10 Alveolata dataset as the reference database [43]. Transcripts were annotated against the KEGG database via DIAMOND, then finally read mapping and quantification was performed (SALMON v.1.4.0). All raw sequences are available on NCBI SRA (PRJNA1125028).

### Statistical analysis

Statistical analysis was conducted in R (v4.2.1). Statistical differences of percent contribution of FA changes between treatments were assessed via ANOVA, with Tukey’s HSD test for multiple comparisons used to find significant differences. Unless otherwise stated in the results, significant differences between treatments had a p value of <0.05. For transcriptomics data, Kegg Ortholog numbers were used to merge annotated transcripts, and differential expressed genes identified via *DESeq2* (wald test and parametric fit). Adjusted p-values were calculated using the Benjami and Hochberg method (BH; [44]). KEGG pathway groups were created for differentially expressed genes and statistically significant log fold changes were tallied and plotted using *ggplot2* [45]. Differentially expressed genes from photosynthetic, FA biosynthesis and FA degradation pathways were mapped against pathway diagrams using the KEGG database. The pipeline for statistical analysis is available on GitHub (https://github.com/JustJaxz/Lipids-and-Light).

## Results

### Growth curves

**Fig 2:**
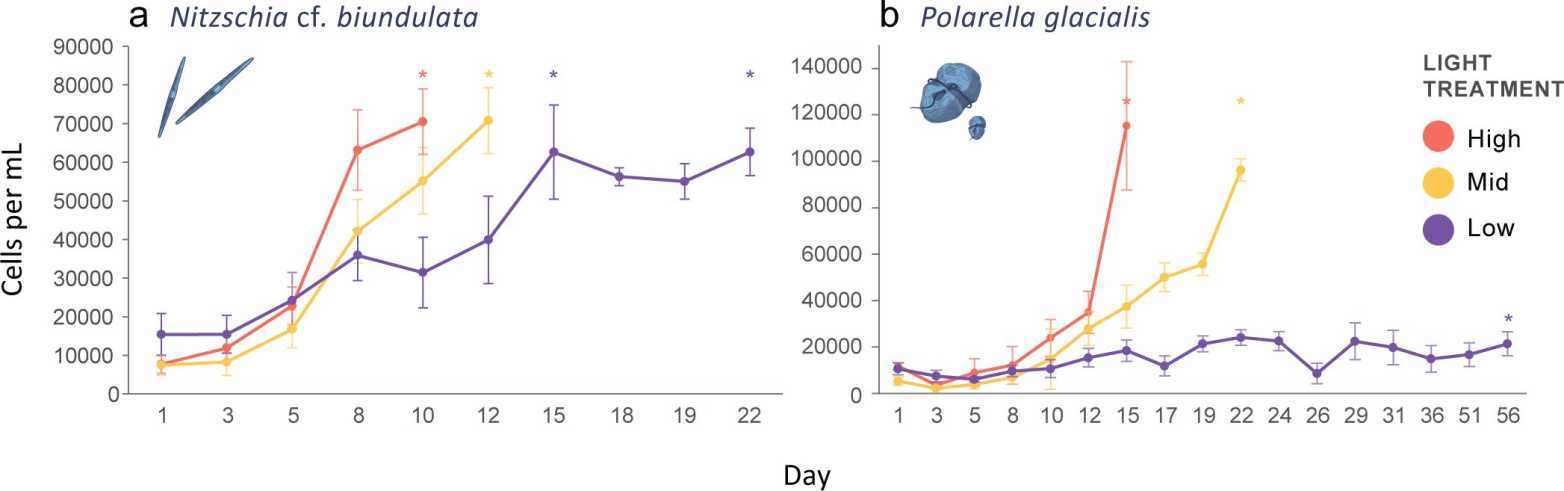
Growth curves for a) *Nitzschia* cf. *biundulata* and b) *Polarella glacialis* under high, mid, and low light experimental conditions. * marks harvest day.

Growth rates for microalgal cultures *N*. cf. *biundulata* and *P*. *glacialis* varied under different light treatments (Fig 2). The high and mid light conditions for *N*. cf. *biundulata* reached the exponential growth phase target range of 60, 000–70,000 cells/mL in 10 and 12 days respectively. The five low light replicates reached the target range at two different time points: 15 days (n = 3) and 22 days (n = 2). *Polarella glacialis* was harvested at 15 days (all high replicates), 22 days (all mid light replicates) and 56 days (all low light replicates). High and mid light *P*. *glacialis* replicates were harvested in the target range of 70,000–100,000 cells/mL, low light replicates did not reach exponential phase targets (average cell density: 21,370 ± 5107 cells/mL).

### Fatty acids (FAs)

**Fig 3:**
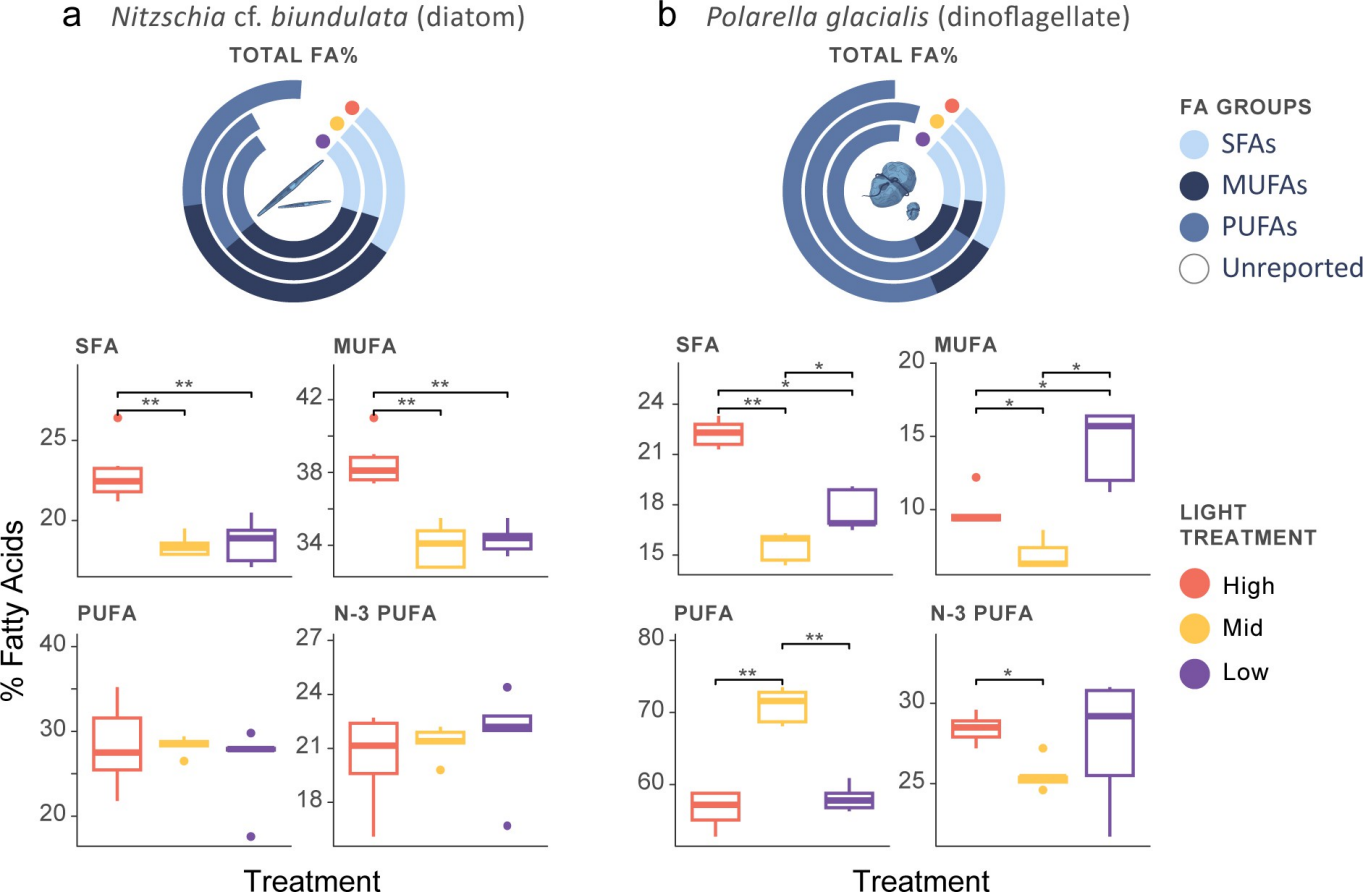
Total fatty acid (FA) profile and percentages of saturated FAs (SFAs), monounsaturated FAs (MUFAs) and polyunsaturated fatty acids (PUFAs) and omega 3 FAs (N-3-PUFA) produced by a) *Nitzschia* cf. *biundulata* and b) *Polarella glacialis* under high, mid, and low light experimental conditions. Boxplots display median values, and significant changes in FA production are annotated: *p = < 0.05, **p = < 0.01, *** p = < 0.001, ****p = < 0.0001 (P value adjustment method: Benjamini-Hochberg).

There were some significant differences in overarching FA groups for *N*. cf. *biundulata* among light treatments. Production of saturated FAs (SFAs) was significantly different (ANOVA: F = 18.6, p < 0.001) with high light producing more than mid (Tukey: 95% CI [-0.31, -0.10], p < 0.001) and low (95% CI [-0.30, -0.09], p < 0.001) treatments (Fig 3A). There was no significant difference between mid and low light for SFAs. Monounsaturated FAs (MUFAs) showed the same trend with significant differences (F = 26.7, p <0.0001) between high and both treatment conditions (95% CI, mid: [-0.17, -0.07] p < 0.01; low [-0.16, -0.06] p < 0.01), with no significant difference between mid and low treatments (Fig 3A). No significant differences were observed in polyunsaturated (PUFA) and Omega-3 polyunsaturated FAs (N-3 PUFA) production for *N*. cf. *biundulata*.

Cultures of *P*. *glacialis* had significant differences in production of SFAs and MUFAs among all experimental conditions (SFAs: F = 59.88, p < 0.001; MUFAs: F = 22.77, p < 0.001) with mid light significantly lower than both high (95% CI, SFA: [-8.4, -5.1], p < 0.01; MUFA: [-5.9, -0.1], p < 0.05) and low light (95% CI, SFA: [-3.84, -0.48], p < 0.05; MUFAs: [-10.3, -4.5], p < 0.05; Fig 3A). High and low light also had significant differences in FA production, with higher SFAs in high light conditions (95% CI, SFA: [-6.3, -2.9], p < 0.05) and higher MUFAs in low light (MUFAs: [1.4, 7.3], p < 0.05; Fig 3B). Proportional contribution of PUFAs was substantially greater than other reported FAs in our results, all *P*. *glacialis* cultures, ranging from 53%– 74% of total FAs (Fig 3B). PUFAs in the mid light condition were produced in significantly higher proportions than both high (95% CI, [10.5, 18.3], p < 0.01) and low light (95% CI, [8.92, 16.72], p < 0.01) conditions. N-3 PUFAs, including Stearidonic and Eicosapentaenoic acids were significantly higher in high light treatments than mid (95% CI, [-6.98, 1.22], p < 0.05). Low light had no significant differences in production from either high or mid conditions.

### Saturated fatty acids (SFAs)

**Fig 4:**
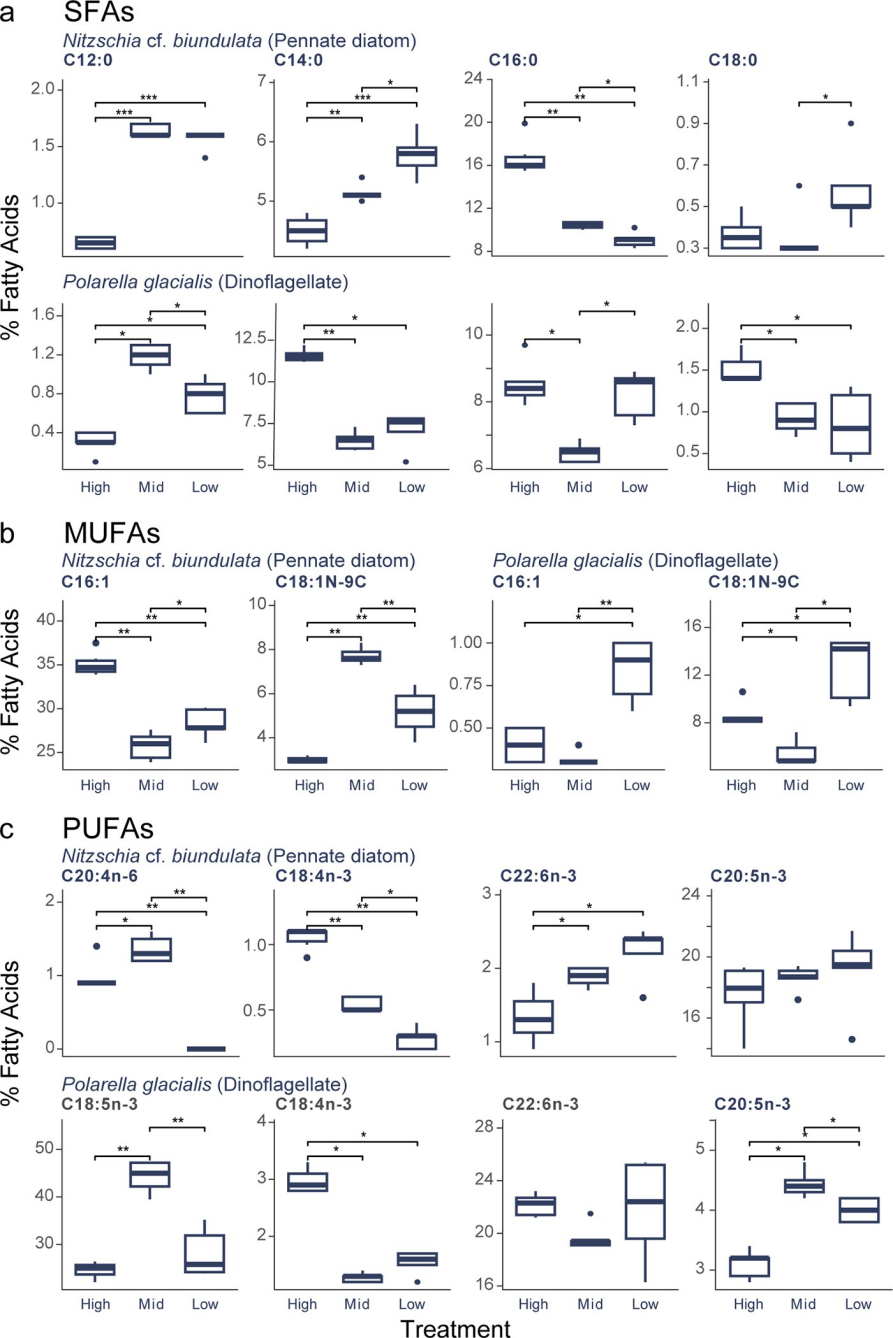
Percentages of a) Saturated Fatty Acids (SFAs), b) monounsaturated fatty acids (MUFAs) and c) polyunsaturated fatty acids (PUFAs) produced by *Nitzschia* cf. *biundulata* and *Polarella glacialis* cultures under high, mid and low light experimental conditions. Results reported in this figure represent FAs that had significant changes or contribute substantially to percent total FAs. Full FA results in S4 Table in S1 File. SFAs include: Lauric (C12:0), Myristic (C14:0), Palmitic (C16:0), Steric (C18:0); MUFAs include: Palmitoleic (C16:1), Oleic (C18:1n-9C); and PUFAs include Arachidonic (C20:4n-6), Stearidonic (C18:4n-3), Docosahexaenoic (C22:6n-3), Eicosapentaenoic (C20:5n-3) and Octadecapentaenoic (C18:5n-3). Boxplots display median values, and significant changes in production are annotated: *p = < 0.05, **p = < 0.01, *** p = < 0.001 (P value adjustment method: Benjamini-Hochberg).

The major SFAs were similar in both *N*. cf. *biundulata* and *P*. *glacialis* isolates (Fig 4, S4 Table in S1 File). In *N*. cf. *biundulata* cultures, lauric acid (C12:0) was significantly lower in high light compared to mid (95% CI [0.42, 0.52] p < 0.01) and low (95% CI [0.39, 0.49], p < 0.01) treatments and myristic acid (C14:0,) significantly increased as light decreased (Low-mid: 95% CI [-0.17, -0.03], p < 0.01; mid-high: [0.04, 0.18], p < 0.01). Palmitic acid (C16:0) significantly decreased as light increased (Low-mid: 95% CI [0.1, 0.24], p < 0.05; mid-high: [-0.55, -0.32], p < 0.0001) and was the most abundant SFA for *N*. cf. *biundulata* cultures. Stearic acid production (C18:0) significantly increased between mid and low conditions (Fig 4A; 95% CI [-0.30, 0.00], p < 0.05).

*Polarella glacialis* had a significant increase in C12:0 between high and both mid (95% CI [0.63, 1.13], p < 0.05) and low (95% CI [0.23, 0.73], p < 0.05) treatments, with mid also producing significantly higher amounts than low light (95% CI [0.15, 0.65], p < 0.05). C14:0 significantly increased in high light compared to other light levels (95% CI Low: [-5.77, -3.23], p < 0.05; mid: [0.63, 1.13], p < 0.01). Production of C16:0 was significantly lower in mid light compared to both high (95% CI [-3.09, -1.07], p < 0.05) and low light (95% CI [-2.75, -0.73], p < 0.05), while C18:0 significantly decreased between high light and both mid (95% CI [-1.06, -0.14], p < 0.05) and low conditions (95% CI [-1.14, -0.22], p < 0.05; Fig 4A).

### Monounsaturated fatty acids (MUFAs)

Two major MUFA profiles were identified in both *N*. cf. *biundulata* and *P*. *glacialis* isolates (Fig 3B). In *N*. cf. *biundulata* cultures palmitoleic acid (C16:1) was significantly lower in mid light conditions than both high (95% CI [-0.38, -0.22], p < 0.001) and low light (95% CI [-0.18, -0.01], p < 0.05). In contrast, the production of oleic acid (C18:1n-9C) in mid light was significantly higher than both high (95% CI [0.62, 0.94], p < 0.01) and low light (95% CI [0.19, 0.53], p < 0.01). *Polarella glacialis* cultures had significantly more C16:1 in low light conditions than high (95% CI [0.23, 0.65], p < 0.05) and mid (95% CI [-2.75, -0.73], p < 0.01). Contribution of C18:1N-9C were significantly lower in mid light than both other conditions (95% CI, low: [-0.73, -0.31], p < 0.05; high: [0.23, 0.65], p < 0.05) (Fig 4B; S4 Table in S1 File).

### Polyunsaturated fatty acids (PUFAs)

Four major PUFAs were detected for *N*. cf. *biundulata* and *P*. *glacialis* (Fig 4C). *N*. cf. *biundulata* had a significant increase in arachidonic acid (C20:4n-6) in mid light conditions compared to high (95% CI [0.06, 0.29], p < 0.05) and low light (95% CI [0.73, 0.98], p < 0.01). Stearidonic (C18:4n-3) percentage decreased with light levels, with highest production of C18:4n-3 in high light and lowest in low light. Both mid and low light produced significantly higher levels of Docosahexaenoic acid (C22:6n-3) than high (95% CI, low: [0.14, 0.51], p < 0.05; mid: [0.04, 0.40], p < 0.05). Eicosapentaenoic (C20:5n-3) contributed substantially to PUFA proportion overall in *N*. cf. *biundulata* and did increase linearly with light levels, however these changes were not statistically significant.

Octadecapentaenoic (C18:5n-3) was only detected in *P*. *glacialis*, contributing between 22%– 47% of total PUFAs. *P*. *glacialis* had significantly higher levels of C18:4n-3 in high conditions compared to both mid (95% CI [-2.00, -1.40], p < 0.05) and low light (95% CI [-1.74, -1.14], p < 0.05). This was significantly higher in mid light conditions compared to both high (95% CI [13.50, 25.82], p < 0.01) and low light (95% CI [9.88, 22.20], p < 0.01). There was no significant difference between high and low light. Mid light also had significantly higher amounts of Eicosapentaenoic (C20:5n-3) than both high (95% CI [0.96, 1.72], p < 0.05) and low light (95% CI [0.06, 0.82], p < 0.05). Docosahexaenoic acid (C22:6n-3) was a relatively large contributor to total PUFAs, ranging between 16–25% across treatments. There were no significant changes in C22:6n-3 production across light treatments (Fig 4C; S4 Table in S1 File).

### Transcriptomics

#### Sequencing output and assembly

Transcriptome assembly for *N*. cf. *biundulata* contained 146,966 transcripts and was 98.9% complete, with 17% single copies, 81.9% duplicated, 0.6% fragmented and 0.5% missing according to BUSCO. For *P*. *glacialis* assembly of the transcriptome contained 2,333,204 transcripts was 96.5% complete, with 45.6% single, 50.9% double, 2.9% fragmented and 0.6% missing according to BUSCO. Significant differences in transcriptomes were found for all experimental conditions of both *N*. cf. *biundulata* (PERMANOVA: F = 25.04; p = 0.001) and *P*. *glacialis* (PERMANOVA: F = 9.42; P = 0.001) cultures (S5 Fig in S1 File).

#### Differentially expressed genes

Low light levels are representative of thick sea ice conditions (~2 m thick). Gene regulation is reported using low light as the baseline, with up or down regulation in relation to low light transcripts. There was a substantial number of differentially expressed genes among each light level treatment for both *N*. cf. *biundulata* and *P*. *glacialis*. For *N*. cf. *biundulata* most of these were related to genetic information processing, environmental information processing and ‘other metabolism’. Briefly, the genetic information processing genes encompass mechanisms involved in DNA replication, transcription and translation, environmental information processing refers to pathways and responses that allow organisms to sense and adapt to changes in their environment. Other metabolism encompasses a variety of metabolic pathways that are not classified under primary metabolic functions such as secondary metabolism and energy production. Lipid metabolism genes among *N*. cf. *biundulata* did have a number of differentially expressed genes, with a substantial portion of those from the FA biosynthesis, elongation, and degradation pathways. A number of energy metabolism, and specifically photosynthesis genes were also differentially expressed (S6 Fig in S1 File).

Overall, there was a lower number of differentially expressed genes for all *P*. *glacialis* light treatments compared to *N*. cf. *biundulata*. Genetic information and environmental information processing also accounted for the majority of differentially expressed genes in *P*. *glacialis*. However, translation genes in low light treatments, energy metabolism and ‘other metabolism’ also had higher numbers when compared to other pathways within *P*. *glacialis* treatments (S6 Fig in S1 File). There were exceptionally low levels of differentially expressed genes involved in lipid metabolism, with none for FA biosynthesis or elongation, and only two involved in the FA degradation pathway.

#### Photosynthetic gene pathway

**Fig 5:**
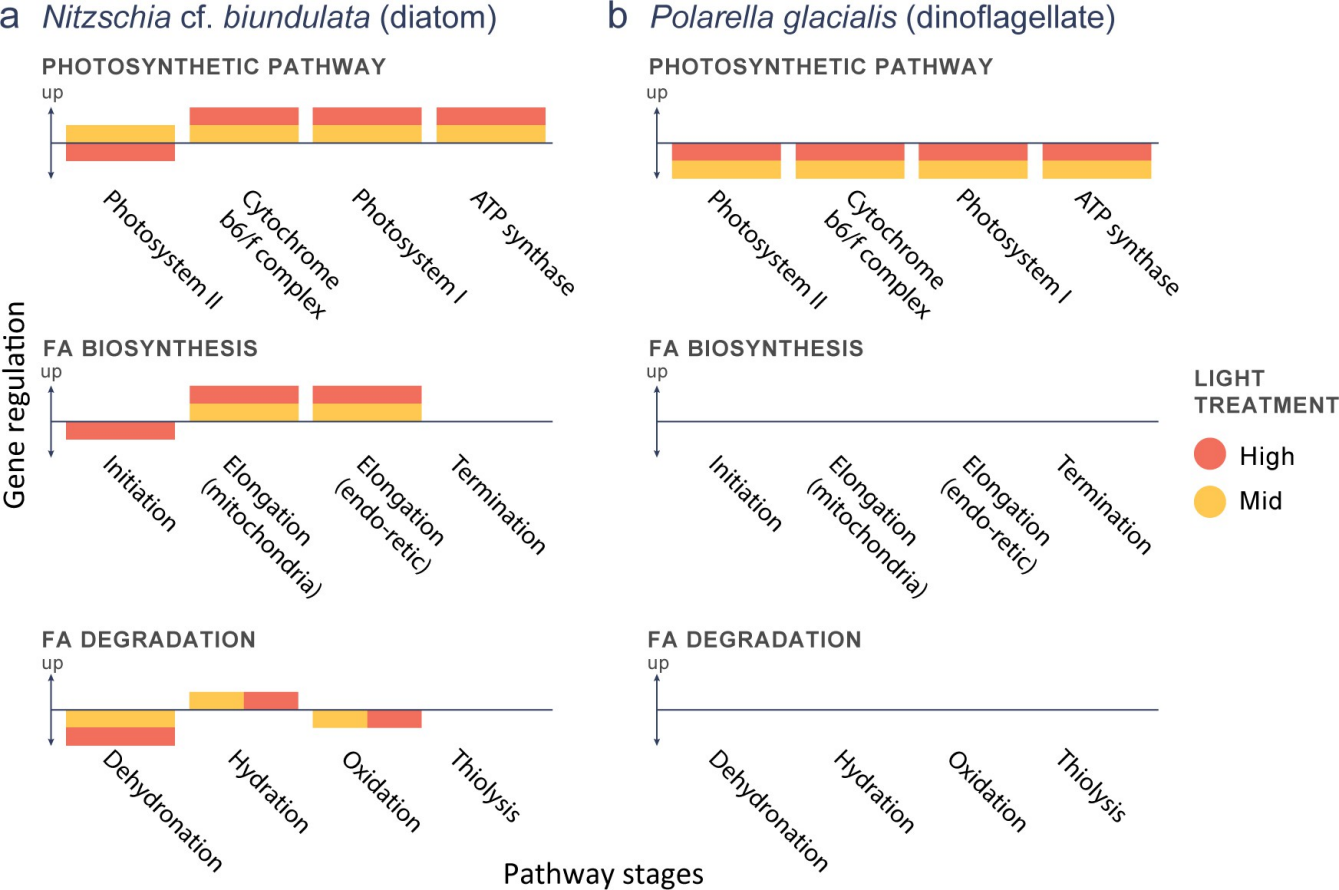
Simplified gene pathways with overarching patterns of differential expression for genes from a) *Nitzschia* cf. *biundulata* and b) *Polarella glacialis* cultures grown in high, mid and low light conditions. Up and down regulation of high and mid light are noted in relation to low light conditions, as these are reflective of ‘typical’ sea ice conditions. When bars are stacked this represents differential expression between high and mid, while side by side indicates no differential expression.

*Nitzschia* cf. *biundulata* had differentially expressed genes across all regions of the photosynthetic pathway (Fig 5A). Mid light conditions caused up-regulation of multiple genes in photosystem II (PSII) while high light conditions caused down-regulation. The PSII pathway showed similar patterns of increased activity in the mid light treatment compared to the low light treatment, although these changes were not statistically significant (S7 Fig in S1 File). Differentially expressed genes within the cytochrome b_6_/f complex (Photosystem electron transport; Pet) were consistently up regulated in mid light conditions. Up-regulation of Photosystem I (PSI) differentially expressed genes were also generally observed in mid light conditions. There was only one differentially expressed gene in ATPase synthesis region, which was also upregulated in mid light conditions. Cultures of *P*. *glacialis* had 22 differentially expressed photosynthesis genes compared to 16 for *N*. cf. *biundulata* (S8 Fig in S1 File). Differentially expressed photosynthesis genes in *P*. *glacialis* were evenly distributed across the PSII, Pet, PSI, and ATPase regions. In the mid-light treatment, gene expression was consistently downregulated across all components of the photosynthetic system (Fig 5B).

#### FA biosynthesis and degradation

Differentially expressed genes from the initiation stage of the FA biosynthesis pathway from *N*. cf. *biundulata* cultures grown in mid light conditions were mostly down-regulated compared to low light (Fig 5A). Up-regulation of genes occurred in both the mitochondrial and endoplasmic reticulum elongation stages of the FA biosynthesis pathway, and no differentially expressed genes were detected in the termination phase. Genes were down-regulated in the dehydrogenation (removal or transfer of a proton, or hydrogen) and oxidation stages of the FA degradation pathway from both mid and high light cultures. However, FA degradation genes related to hydration were up regulated for both mid and high light (S9-S11 Figs in S1 File). There were no differentially expressed genes observed throughout both biosynthesis, elongation, and the main FA degradation pathways in *P*. *glacialis* cultures (Fig 5B; S12 Fig in S1 File). The two involved in FA degradation processes that were differentially expressed were in the aldehyde to FA component of the pathway.

## Discussion

Light levels emulating thinning sea ice conditions caused substantially different intracellular responses in microalgal isolates from two high-level taxonomic groups. Both the pennate diatom (*N*. cf. *biundulata)* and dinoflagellate (*P*. *glacialis)* cultures experienced changes in growth rates, as well as shifts in FA composition and gene expression in energy and FA metabolism pathways. Growth rates increased for both isolates as light intensity increased. Low light conditions were clearly unfavourable for *P*. *glacialis* growth, as cell densities barely increased, and all cultures failed to enter the exponential growth phase during the experiment. This inability for *P*. *glacialis* to reach exponential growth under low light conditions was surprising, as this dinoflagellate has been reported as a widespread and resilient species in polar sea ice environments where light levels are often low [46, 47]. The poor growth under low light could be due to several factors, such as not reaching the minimal light threshold to support efficient photosynthesis, day-length or light wavelength cues which may not have been fully replicated in the experimental set up.

Additionally, photosynthetic genes were down-regulated in high light treatments compared to low light, indicating *P*. *glacialis* growing under low light was investing considerable energy in survival as opposed to growth. Optimal growth conditions related to light are not uniform for microalgae taxa [11]. For example, Arctic sea ice algae are less tolerant of high light levels and may experience photoinhibition, a process where excessive light energy damages photosynthetic apparatus [48, 49]. In contrast, higher light is linked to increased cell densities and faster growth rates [50–52], as observed here. The Low treatment light levels used in this study are in the lower range of intensities investigated previously (Low: 1.5 ± 1, Mid: 10 ± 1, High: 90 ± 1 μmol photons m^−2^ s^−1^) [e.g. 53–55]. Given that both isolate species are naturally exposed to large changes in light intensity in Antarctic sea ice environments annually [4], all the light levels used in this study fall within the range they would naturally experience.

FA profiles for *N*. cf. *biundulata* showed no significant changes in production within the overarching groups of SFAs, MUFAs and PUFAs. Though a *N*. cf. *biundulata* FA profile has not previously been characterised, other *Nitzschia* sp. profiles are comparable to those observed here under high light conditions [56]. However, variation among *Nitzschia* species are often greater than differences observed between light treatments in this study. C20:5N-3 made up a substantial proportion of *N*. cf. *biundulata*’s PUFAs, similar to other *Nitzschia* sp. profiles [56]. The indication of a linear decrease in EPA with rising light levels, though not statistically significant, suggests that *N*. cf. *biundulata* may tend to produce less C20:5N-3 under high light conditions. This reduction of C20:5n-3 in microalgae grown under increased irradiance has been observed in several other microalgal species [57], including *Nitzschia alexandrina* [58]. C20:5n-3 is a major structural component in chloroplast membranes [59, 60], the reduction under high irradiance may indicate a broader metabolic shift from production of structural (PUFA) to storage lipids [57]. This aligns with the increase in production of C16:1 and C16:1n-7 observed here in *N*. cf. *biundulata* grown under high irradiance. Each of these can be important in the biosynthesis of the primary form of storage lipids, triacylglycerols (TAGs) [61]. However, the lack of statistical significance means this trend cannot be confidently linked to light changes without further investigation.

Significant changes within individual SFA production for our *N*. cf. *biundulata* cultures included the reduction of C14:0, which would increase cell membrane fluidity, allowing for enhanced photosynthetic efficiency and reduction in the risk of photodamage [62, 63]. Increased fluidity may also help prevent damage to membranes due to the formation of ice crystals [64]. This response, coupled with the up-regulation of photosynthetic genes indicates *N*. cf. *biundulata* cells are enhancing photosynthetic activity and biomass production [65, 66] in light conditions emulating thinning sea ice conditions. The elevation of C16:0 in conjunction with MUFA C18:1N-9C in mid light would suggest an increase in energy storage [66, 67]. Interestingly, in our highest light treatments, we observed elevated levels of C16:1, which aligns with findings from [68, 69] that suggest increased C16:1 is indicative of lipid accumulation under high irradiance and nutrient stress. This increase in energy storage is reinforced by the down-regulation of FA degradation genes in mid-light cultures [70].

FA profiles for *P*. *glacialis* cultures were dominated by PUFAs, and mainly C18:5N-3 which contributed up to 50% of total FAs, aligning with previous FA profiles for this species [71]. Significant differences in the proportion of SFAs, MUFAs, PUFAs and N-3 PUFAs relative to total FAs were observed. In mid light, the proportion of SFAs and MUFAs decreased, while the proportion of PUFAs increased compared to both low and high light conditions. This most likely indicates a change in energy storage strategy [66, 72]. For *P*. *glacialis*, the significant changes in PUFA proportions were marked by a decrease under both low and high light conditions. A reduction in PUFA levels is commonly seen under lower light [72, 73], making the lower FA production levels in conditions with the highest light intensity intriguing.

The unimodal response of PUFA proportion to light treatments may be related to a species-specific optimum for *P*. *glacialis*. A draft genome assembly of *P*. *glacialis* strains has highlighted functional innovation associated with environmental adaptation and niche specialisation [74]. Higher PUFA production in mid light cultures may indicate increased thylakoid membrane stacking for photosynthesis optimisation [75]. PUFAs are important for maintaining membrane fluidity which supports the formation of stacked membranes, particularly under ideal light conditions [76]. This unimodal pattern could also reflect a trade-off between energy capture and photoprotection, where intermediate light levels allow for optimal energy production without triggering excessive stress responses, from too little or too much light.

Interestingly, this was not reinforced in photosynthetic pathway transcripts, as photosynthetic genes in the mid-light treatment were down regulated in comparison to low-light. This difference in responses may be explained by a shift in resource allocation towards protective mechanisms or increased metabolic efficiency under mid-light conditions. In contrast, the up-regulation of photosynthetic genes and reduction in PUFA proportion in low-light treatments indicates an attempt to decrease proton leakage and improve energy efficiency and production within the cells [65, 77]. Similar to *N*. cf. *biundulata* cultures in this study the reduction of PUFA proportion in high light may indicate the prioritisation of photoprotective strategies [29]. This is achieved by reduced production of structural lipids (PUFAs) as fewer chloroplast membranes are needed under bright light [57, 60].

High levels of C18:5N-3 have been observed in *P*. *glacialis* previously [71], however there was a significant increase in production of C18:5N-3 (up to 20%) under emulated thinning ice conditions. Biologically high levels of C18:5N-3 indicates enhanced membrane fluidity, and improved synthesis of longer-chain PUFAs like C20:5N-3 and C22:6N-3 [78, 79]. No evidence of up-regulation within FA biosynthesis pathways was found to suggest an effort to increase synthesis in mid light. Moreover, no significant increase was observed in C22:6N-3. C20:5N-3 did increase in production in mid light conditions, though compared to C22:6N-3 (19–22%) and C18:5N-3 (25–44%) was only a minor contributor to overall PUFA production (3–5%). This observation of biomolecule production without corresponding transcriptomic changes is not unique to *P*. *glacialis*. The dinoflagellate species *Alexandrium catenella* and *A*. *minutum* have both demonstrated that their production of toxic biomolecules may also not be regulated at the transcriptional level [80, 81]. These observations suggest that dinoflagellates employ different strategies compared to diatoms when acclimatising to varying conditions, highlighting the complexity of metabolic regulation in these organisms.

The FA C18:5N-3 is an ichthyotoxic molecule, (toxic to fish) [82, 83]. Other marine dinoflagellates that are high producers of C18:5N-3 have been linked to fish mortality [82, 84]. Increased production of C18:5N-3 under emulated thinning ice conditions could be of concern for toxicity at higher trophic levels in polar environments. Further investigation into the influence of other abiotic changes in conjunction with light would be required to elucidate the level of risk this species may present in the future.

Changes in sea ice cover and duration in McMurdo Sound will influence microalgae community composition and potentially reduce diversity [6]. Predicted shifts within Antarctic microalgae communities, moving from larger diatom species toward smaller taxa like *P*. *glacialis*, could alter energy transfer efficiency within Antarctic food webs, affecting overall productivity [85–88]. Our findings indicate that *P*. *glacialis* enhances C18:5N-3 production under mid-light conditions, which raises concerns about the potential emerging threat of toxins in the Antarctic food web and the associated risks to marine fauna. Conversely, *N*. cf. *biundulata* exhibited stable fatty acid profiles but reduced specific SFAs, a change that could enhance membrane fluidity and photosynthetic efficiency. This response might allow *N*. cf. *biundulata* to acclimate effectively to fluctuating light conditions, ensuring metabolic efficiency amidst environmental changes.

The transfer of energy and carbon through trophic levels in the Antarctic food webs is anticipated to be impacted by reduced microalgal community diversity [85, 86]. Smaller cell sizes reduce grazing efficiency for primary consumers [89, 90]; combined with lower diversity, this means shifts in FA and other biomolecule production could be amplified within ecosystems. Anticipated shifts in community composition and trophic transfer underscore the need to understand single stressor impacts, as examined in our study, to better predict the multifaceted effects of climate change on Antarctic ecosystems.

Lab based experiments to assess the impact of environmental changes have limitations. This study only includes a single stressor, which is not reflective of what will be experienced in the natural environment [e.g. 50, 91]. Understanding the acclimation response of species to single stressors does still provide valuable insights, such as establishing baseline responses, identifying key molecular and physiological mechanisms, or determining stress tolerance thresholds. Prolonged growth of microalgae isolates in culture conditions may also influence the acclimation response of both species to lower light levels [92]. Future work investigating the community level biomolecular composition and meta-transcriptomics *in situ* would help elucidate how much impact variable light levels will have on nutrient and carbon transfer within the sea ice and sub-ice platelets ecosystems. Additionally, the use of respective data such as absolute fatty acid amounts or POC standardised fatty acid content.

Light levels emulating thinning sea ice and snow conditions have an impact on the photosynthetic activity and FA composition of *N*. cf. *biundulata* and *P*. *glacialis* isolates. Though light level changes are not necessarily a concern with changing climate in most marine ecosystems, thinning of ice and variation of snow loads will impact light condition in sea ice environments [5]. Differing responses in photosystems and growth rates have the potential to create changes in overall microalgae community composition and their nutritional quality with potential impacts on the whole food web. Further studies are needed to assess the resulting change in macronutrients available to higher trophic levels and food web structure in Antarctica.

## Supporting information

S1 File Comprehensive phylogenetic, microscopy, experimental conditions, fatty acid analysis, and transcriptomic data for *Nitzschia cf*. *biundulata* and *Polarella glacialis* under varying light treatments. (PDF)

S1 Graphical abstract (TIF)

## Abbreviations

C20:4N-6: Arachidonic Acid
C22:6N-3: Docosahexaenoic Acid
C20:5N-3: Eicosapentaenoic Acid
FA: Fatty Acid
GC: Gas Chromatography
C12:0: Lauric Acid
C14:0: Myristic Acid
MUFA: Monounsaturated Fatty Acid
C18:1N-9C: Oleic Acid
C18:5N-3: Octadecapentaenoic Acid
C16:0: Palmitic Acid
C16:1: Palmitoleic Acid
PUFA: Polyunsaturated Fatty Acid
C18:0: Steric Acid
C18:4N-3: Stearidonic Acid
SFA: Saturated Fatty Acid

## References

[1] Arrigo K.R., Sea ice as a habitat for primary producers. Sea Ice, ed. Thomas D. 2017, Oxford, Uk: Wiley-Blackwell.

[2] Lizotte M.P., The Contributions of Sea Ice Algae to Antarctic Marine Primary Production. American Zoologist, 2001. 41(1): p. 57–73.

[3] Bernard K.S., et al., The contribution of ice algae to the winter energy budget of juvenile Antarctic krill in years with contrasting sea ice conditions. ICES J. Mar. Sci., 2019. 76: p. 206–216.

[4] Thomas D.N. and Dieckmann G.S., Antarctic Sea ice—a habitat for extremophiles. Science, 2002. 295(5555): p. 641–4. doi: 10.1126/science.1063391 11809961

[5] Meredith M., et al., Polar Regions, in IPCC Special Report on the Ocean and Cryosphere in a Changing Climate, Pörtner H.O, et al., Editors. 2019, IPCC: Cambridge, UK and New York, USA. p. 203–320.

[6] Stuart J., et al., A glimpse into the future? How the timing of sea ice formation influences associated microalgal communities. Submitted. 10.1016/j.isci.2025.112417 PMC12063147 40352721

[7] Silva S.C., Ferreira I., Dias M.M., and Barreiro M.F., Microalgae-Derived Pigments: A 10-Year Bibliometric Review and Industry and Market Trend Analysis. Molecules, 2020. 25(15). doi: 10.3390/molecules25153406 32731380 PMC7435790

[8] Minhas A.K., Hodgson P., Barrow C.J., and Adholeya A., A Review on the Assessment of Stress Conditions for Simultaneous Production of Microalgal Lipids and Carotenoids. Front Microbiol, 2016. 7: p. 546. doi: 10.3389/fmicb.2016.00546 27199903 PMC4853371

[9] Zhang Z., et al., A new paradigm for producing astaxanthin from the unicellular green alga Haematococcus pluvialis. Biotechnol Bioeng, 2016. 113(10): p. 2088–99. doi: 10.1002/bit.25976 27563850 PMC5071682

[10] Abiusi F., Wijffels R.H., and Janssen M., Doubling of Microalgae Productivity by Oxygen Balanced Mixotrophy. ACS Sustainable Chemistry & Engineering, 2020. 8(15): p. 6065–6074.

[11] Maltsev Y., Maltseva K., Kulikovskiy M., and Maltseva S., Influence of Light Conditions on Microalgae Growth and Content of Lipids, Carotenoids, and Fatty Acid Composition. Biology (Basel), 2021. 10(10). doi: 10.3390/biology10101060 34681157 PMC8533579

[12] Arrigo K.R., Sea ice ecosystems. Ann Rev Mar Sci, 2014. 6: p. 439–67. doi: 10.1146/annurev-marine-010213-135103 24015900

[13] Perovich D.K., Sea ice and sunlight, in Sea Ice, Thomas D.N., Editor. 2016. p. 110–137.

[14] Zwally H.J., Parkinson C.L., and Comiso J.C., Variability of Antarctic sea ice: and changes in carbon dioxine. Science, 1983. 220: p. 1005–1012. 17754532 10.1126/science.220.4601.1005

[15] Jeffries M.O., Krouse H.R., Hurst-Cushing B., and Maksym T., Snow-ice accretion and snow-cover depletion on Antarctic first-year sea-ice floes. Annals of Glaciology, 2017. 33: p. 51–60.

[16] Eayrs C., Li X., Raphael M.N., and Holland D.M., Rapid decline in Antarctic sea ice in recent years hints at future change. Nature Geoscience, 2021. 14(7): p. 460–464.

[17] Smetacek V. and Nicol S., Polar ocean ecosystems in a changing world. Nature, 2005. 437(7057): p. 362–8. doi: 10.1038/nature04161 16163347

[18] Meiners K.M. and Michel C., Dynamics of nutrients, dissolved organic matter and exopolymers in sea ice, in Sea Ice. 2017. p. 415–432.

[19] Bhavya P.S., et al., A Review on the Macromolecular compositions of phytoplankton and the implications for aquatic biogeochemistry. Ocean Sci., 2018. 54: p. 1–14.

[20] Haberman K.L., Quetin L.B., and Ross R.M., Diet of the Antarctic krill (Euphausia superba Dana). Journal of Experimental Marine Biology and Ecology, 2003. 283(1–2): p. 79–95.

[21] Lee R., Hagen W., and Kattner G., Lipid storage in marine zooplankton. Mar. Ecol. Prog. Ser., 2006. 307: p. 273–306.

[22] Parrish C.C., Lipids in Marine Ecosystems. ISRN Oceanography, 2013. 2013: p. 1–16.

[23] Lang I., Hodac L., Friedl T., and Feussner I., Fatty acid profiles and their distribution patterns in microalgae: a comprehensive analysis of more than 2000 strains from the SAG culture collection. BMC Plant Biol, 2011. 11: p. 124. doi: 10.1186/1471-2229-11-124 21896160 PMC3175173

[24] Cañavate J.P., Advancing assessment of marine phytoplankton community structure and nutritional value from fatty acid profiles of cultured microalgae. Reviews in Aquaculture, 2018. 11(3): p. 527–549.

[25] Finkel Z.V., et al., Phylogenetic Diversity in the Macromolecular Composition of Microalgae. PLoS ONE, 2016. 11: p. e0155977. doi: 10.1371/journal.pone.0155977 27228080 PMC4882041

[26] Saggiomo M., et al., Spring-time dynamics of diatom communities in landfast and underlying platelet ice in Terra Nova Bay, Ross Sea, Antarctica. Journal of Marine Systems, 2017. 166: p. 26–36.

[27] Jonasdottir S.H., Fatty Acid Profiles and Production in Marine Phytoplankton. Mar Drugs, 2019. 17(3). doi: 10.3390/md17030151 30836652 PMC6471065

[28] Gleitz M. and Kirst G.O., Photosynthesis-irradiance relationships and carbon metabolism of different ice algal assemblages collected from Weddell Sea pack ice during austral spring (EPOS 1). Polar Biology, 1991. 11(6).

[29] Mock T. and Kroon B.M., Photosynthetic energy conversion under extreme conditions—II: the significance of lipids under light limited growth in Antarctic sea ice diatoms. Phytochemistry, 2002. 61(1): p. 53–60. doi: 10.1016/s0031-9422(02)00215-7 12165302

[30] Cade-Menun B.J. and Paytan A., Nutrient temperature and light stress alter phosphorus and carbon forms in culture-grown algae. Marine Chemistry, 2010. 121(1–4): p. 27–36.

[31] Xu D., et al., Long-term experiment on physiological responses to synergetic effects of ocean acidification and photoperiod in the Antarctic sea ice algae Chlamydomonas sp. ICE-L. Environ Sci Technol, 2014. 48(14): p. 7738–46. doi: 10.1021/es404866z 24922067

[32] Thomson P.G., McMinn A., Kiessling I., Watson M., and Goldsworthy P.M., Composition and succession of dinoflagellates and chrysophytes in the upper fast ice of Davis Station, East Antarctica. Polar Biology, 2005. 29(4): p. 337–345.

[33] Guillard R.R.L., Culture of Phytoplankton for Feeding Marine Invertebrates, in Culture of Marine Invertebrate Animals, Smith W.Land Chanley M.H, Editors. 1975, Springer: Boston. p. 29–60.

[34] Kihika J.K., et al., Cryopreservation of six Symbiodiniaceae genera and assessment of fatty acid profiles in response to increased salinity treatments. Sci Rep, 2022. 12(1): p. 12408. doi: 10.1038/s41598-022-16735-w 35859115 PMC9300622

[35] Masood A., Stark K.D., and Salem N. Jr., A simplified and efficient method for the analysis of fatty acid methyl esters suitable for large clinical studies. J Lipid Res, 2005. 46(10): p. 2299–305. doi: 10.1194/jlr.D500022-JLR200 16061957

[36] MacManes M.D., The Oyster River Protocol: a multi-assembler and kmer approach for de novo transcriptome assembly. PeerJ, 2018. 6: p. e5428. doi: 10.7717/peerj.5428 30083482 PMC6078068

[37] Bolger A.M., Lohse M., and Usadel B., Trimmomatic: a flexible trimmer for Illumina sequence data. Bioinformatics, 2014. 30(15): p. 2114–20. doi: 10.1093/bioinformatics/btu170 24695404 PMC4103590

[38] Song L. and Florea L., Rcorrector: efficient and accurate error correction for Illumina RNA-seq reads. Gigascience, 2015. 4: p. 48. doi: 10.1186/s13742-015-0089-y 26500767 PMC4615873

[39] Robertson G., et al., De novo assembly and analysis of RNA-seq data. Nat Methods, 2010. 7(11): p. 909–12. doi: 10.1038/nmeth.1517 20935650

[40] Grabherr M.G., et al., Full-length transcriptome assembly from RNA-Seq data without a reference genome. Nat Biotechnol, 2011. 29(7): p. 644–52. doi: 10.1038/nbt.1883 21572440 PMC3571712

[41] Bankevich A., et al., SPAdes: a new genome assembly algorithm and its applications to single-cell sequencing. J Comput Biol, 2012. 19(5): p. 455–77. doi: 10.1089/cmb.2012.0021 22506599 PMC3342519

[42] Seppey M., Manni M., and Zdobnov E.M., BUSCO: Assessing Genome Assembly and Annotation Completeness. Methods Mol Biol, 2019. 1962: p. 227–245. doi: 10.1007/978-1-4939-9173-0_14 31020564

[43] Hart A.J., et al., EnTAP: Bringing faster and smarter functional annotation to non-model eukaryotic transcriptomes. Mol Ecol Resour, 2020. 20(2): p. 591–604. doi: 10.1111/1755-0998.13106 31628884

[44] Love M.I., Huber W., and Anders S., Moderated estimation of fold change and dispersion for RNA-seq data with DESeq2. Genome Biol, 2014. 15(12): p. 550. doi: 10.1186/s13059-014-0550-8 25516281 PMC4302049

[45] Wickham H., ggplot2: Elegant Graphics for Data Analysis. 2016, New York: Springer-Verlag.

[46] Montresor M., Procaccini G., and Stoecker D.K., Polarella Glacialis, Gen. Nov., Sp. Nov. (Dinophyceae): Suessiaceae Are Still Alive! J. Phycol., 1999. 35(1): p. 186–197.

[47] Montresor M., Lovejoy C., Orsini L., Procaccini G., and Roy S., Bipolar distribution of the cyst-forming dinoflagellate Polarella glacialis. Polar Biol., 2003. 26(3): p. 186–194.

[48] Leu E., Wiktor J., Søreide J.E., Berge J., and Falk-Petersen S., Increased irradiance reduces food quality of sea ice algae. Marine Ecology Progress Series, 2010. 411: p. 49–60.

[49] Kvernvik A.C., et al., Arctic sea ice algae differ markedly from phytoplankton in their ecophysiological characteristics. Marine Ecology Progress Series, 2021. 666: p. 31–55.

[50] Hoppe C.J.M., et al., Resistance of Arctic phytoplankton to ocean acidification and enhanced irradiance. Polar Biol, 2018. 41(3): p. 399–413. doi: 10.1007/s00300-017-2186-0 31983801 PMC6952045

[51] Richardson K., Beardall J., and Raven J.A., Adaptation of Unicellular Algae to Irradiance: An Analysis of Strategies. New Phytologist, 2006. 93(2): p. 157–191.

[52] Trimborn S., et al., Two Southern Ocean diatoms are more sensitive to ocean acidification and changes in irradiance than the prymnesiophyte Phaeocystis antarctica. Physiol Plant, 2017. 160(2): p. 155–170. doi: 10.1111/ppl.12539 28019019

[53] Treves H., et al., A newly isolated Chlorella sp. from desert sand crusts exhibits a unique resistance to excess light intensity. FEMS Microbiol Ecol, 2013. 86(3): p. 373–80. doi: 10.1111/1574-6941.12162 23773145

[54] Van Wagenen J., et al., Effects of Light and Temperature on Fatty Acid Production in Nannochloropsis Salina. Energies, 2012. 5(3): p. 731–740.

[55] Wu M., et al., Effects of different abiotic stresses on carotenoid and fatty acid metabolism in the green microalga Dunaliella salina Y6. Annals of Microbiology, 2020. 70(1).

[56] Mitani E., et al., Fatty acid composition profiles of 235 strains of three microalgal divisions within the NIES Microbial Culture Collection. Microb. Resour. Syst, 2017. 33(1): p. 19–29.

[57] Fábregas J., Maseda A., Domínguez A., and Otero A., The cell composition of Nannochloropsis sp. changes under different irradiances in semicontinuous culture. World Journal of Microbiology and Biotechnology, 2004. 20(1): p. 31–35.

[58] Cointet E., et al., Effects of light and nitrogen availability on photosynthetic efficiency and fatty acid content of three original benthic diatom strains. PLoS One, 2019. 14(11): p. e0224701. doi: 10.1371/journal.pone.0224701 31694047 PMC6834396

[59] Kates M. and Volcani B.E., Lipid components of diatoms. Biochim Biophys Acta, 1966. 116(2): p. 264–78. doi: 10.1016/0005-2760(66)90009-9 5956913

[60] Cohen Z., Vonshak A., and Richmond A., Effect of Environmental Conditions on Fatty Acid Composition of the Red Alga Porphyridium Cruentum: Correlation to Growth Rate1. Journal of Phycology, 2008. 24(3): p. 328–332.

[61] Chen G., Jiang Y., and Chen F., Fatty acid and lipid class composition of the eicosapentaenoic acid-producing microalga, Nitzschia laevis. Food Chemistry, 2007. 104(4): p. 1580–1585.

[62] Guihéneuf F., Mimouni V., Ulmann L., and Tremblin G., Combined effects of irradiance level and carbon source on fatty acid and lipid class composition in the microalga Pavlova lutheri commonly used in mariculture. Journal of Experimental Marine Biology and Ecology, 2009. 369(2): p. 136–143.

[63] Kurima K., et al., High Myristic Acid in Glycerolipids Enhances the Repair of Photodamaged Photosystem II under Strong Light. Plant Cell Physiol, 2024. 65(5): p. 790–797. doi: 10.1093/pcp/pcae021 38441322 PMC11138363

[64] Morgan-Kiss R.M., Priscu J.C., Pocock T., Gudynaite-Savitch L., and Huner N.P., Adaptation and acclimation of photosynthetic microorganisms to permanently cold environments. Microbiol Mol Biol Rev, 2006. 70(1): p. 222–52. doi: 10.1128/MMBR.70.1.222-252.2006 16524924 PMC1393254

[65] Quigg A., Kevekordes K., Raven J.A., and Beardall J., Limitations on microalgal growth at very low photon fluence rates: the role of energy slippage. Photosynth Res, 2006. 88(3): p. 299–310. doi: 10.1007/s11120-006-9052-1 16691367

[66] Guschina I.A. and Harwood J.L., Lipids and lipid metabolism in eukaryotic algae. Prog Lipid Res, 2006. 45(2): p. 160–86. doi: 10.1016/j.plipres.2006.01.001 16492482

[67] Arisz S.A., van Himbergen J.A., Musgrave A., van den Ende H., and Munnik T., Polar glycerolipids of Chlamydomonas moewusii. Phytochemistry, 2000. 53(2): p. 265–270. doi: 10.1016/s0031-9422(99)00505-1 10680181

[68] Duncan R.J., et al., Seasonal environmental transitions and metabolic plasticity in a sea-ice alga from an individual cell perspective. Sci Rep, 2024. 14(1): p. 14984. doi: 10.1038/s41598-024-65273-0 38951587 PMC11217269

[69] Leu E., et al., Spatial and Temporal Variability of Ice Algal Trophic Markers—With Recommendations about Their Application. Journal of Marine Science and Engineering, 2020. 8(9).

[70] Poong S.-W., et al., Transcriptome sequencing of an Antarctic microalga, Chlorella sp. (Trebouxiophyceae, Chlorophyta) subjected to short-term ultraviolet radiation stress. Journal of Applied Phycology, 2017. 30(1): p. 87–99.

[71] Thomson P.G., et al., Antarctic Distribution, Pigment and Lipid Composition, and Molecular Identification of the Brine Dinoflagellate Polarella Glacialis (Dinophyceae)1. Journal of Phycology, 2004. 40(5): p. 867–873.

[72] Wacker A., Piepho M., Harwood J.L., Guschina I.A., and Arts M.T., Light-Induced Changes in Fatty Acid Profiles of Specific Lipid Classes in Several Freshwater Phytoplankton Species. Front Plant Sci, 2016. 7: p. 264. doi: 10.3389/fpls.2016.00264 27014290 PMC4792871

[73] Pronina N., Rogova N., Klyachko-Gurvich G., and Furnadzhieva S., Effect of CO 2 concentration on the fatty acid composition of lipids in Chlamydomonas reinhardtii cia-3, a mutant deficient in CO 2-concentrating mechanism. Russian journal of plant physiology, 1998. 45(4): p. 447–455.

[74] Stephens T.G., et al., Genomes of the dinoflagellate Polarella glacialis encode tandemly repeated single-exon genes with adaptive functions. BMC Biol, 2020. 18(1): p. 56. doi: 10.1186/s12915-020-00782-8 32448240 PMC7245778

[75] Sakshaug E., et al., Parameters of photosynthesis: definitions, theory and interpretation of results. Journal of Plankton Research, 1997. 19(11): p. 1637–1670.

[76] Williams W.P., The Physical Properties of Thylakoid Membrane Lipids and Their Relation to Photosynthesis, in Lipids in Photosynthesis: Structure, Function and Genetics, Paul-André S and Norio M, Editors. 2004, Springer, Dordrecht. p. 103–118.

[77] Raven J.A., Kübler J.E., and Beardall J., Put out the light, and then put out the light. Journal of the Marine Biological Association of the United Kingdom, 2000. 80(1): p. 1–25.

[78] Blasio M. and Balzano S., Fatty Acids Derivatives From Eukaryotic Microalgae, Pathways and Potential Applications. Front Microbiol, 2021. 12: p. 718933. doi: 10.3389/fmicb.2021.718933 34659147 PMC8511707

[79] Qi B., et al., Identification of a cDNA encoding a novel C18-Δ9 polyunsaturated fatty acid-specific elongating activity from the docosahexaenoic acid (DHA)-producing microalga, Isochrysis galbana. FEBS letters, 2002. 510(3): p. 159–165. 11801246 10.1016/s0014-5793(01)03247-1

[80] Perini F., et al., SxtA and sxtG gene expression and toxin production in the Mediterranean Alexandrium minutum (Dinophyceae). Mar Drugs, 2014. 12(10): p. 5258–76. doi: 10.3390/md12105258 25341029 PMC4210898

[81] Wiese M., Murray S.A., Alvin A., and Neilan B.A., Gene expression and molecular evolution of sxtA4 in a saxitoxin producing dinoflagellate Alexandrium catenella. Toxicon, 2014. 92: p. 102–12. doi: 10.1016/j.toxicon.2014.09.015 25301480

[82] Sola F., et al., Toxicity of fatty acid 18:5n3 from Gymnodinium cf. mikimotoi: I. morphological and biochemical aspects onDicentrarchus labrax gills and intestine. Journal of Applied Toxicology, 1999. 19(4): p. 279–284. doi: 10.1002/(sici)1099-1263(199907/08)19:4&lt;279::aid-jat579&gt;3.0.co;2-x 10439343

[83] Fossat B., et al., Toxicity of fatty acid 18:5n3 from Gymnodinium cf. mikimotoi: II. intracellular pH and K+ uptake in isolated trout hepatocytes. Journal of Applied Toxicology, 1999. 19(4): p. 275–278. doi: 10.1002/(sici)1099-1263(199907/08)19:4&lt;275::aid-jat578&gt;3.0.co;2-b 10439342

[84] Yasumoto T., et al., Screening for hemolytic and ichthyotoxic components of Chrysochromulina polylepis and Gyrodinium aureolum from Norwegian coastal waters. Toxic Marine Phytoplankton, 1990: p. 436–440.

[85] Duncan R.J. and Petrou K., Biomolecular Composition of Sea Ice Microalgae and Its Influence on Marine Biogeochemical Cycling and Carbon Transfer through Polar Marine Food Webs. Geosciences, 2022. 12(1).

[86] Moline M.A., Claustre H., Frazer T.K., Schofield O., and Vernet M., Alteration of the food web along the Antarctic Peninsula in response to a regional warming trend. Global Change Biology, 2004. 10(12): p. 1973–1980.

[87] Montes-Hugo M., et al., Recent changes in phytoplankton communities associated with rapid regional climate change along the western Antarctic Peninsula. Science, 2009. 323(5920): p. 1470–3. doi: 10.1126/science.1164533 19286554

[88] Neeley A.R., Harris L.A., and Frey K.E., Unraveling Phytoplankton Community Dynamics in the Northern Chukchi Sea Under Sea‐Ice‐Covered and Sea‐Ice‐Free Conditions. Geophysical Research Letters, 2018. 45(15): p. 7663–7671.

[89] Boyd C.M., Heyraud M., and Boyd C.N., Feeding of the Antarctic Krill Euphausia Superba. Journal of Crustacean Biology, 1984. 4(5): p. 123–141.

[90] Quetin L.B. and Ross R.M., Feeding by Antarctic Krill, Euphausia superba: Does Size Matter?, in Antarctic Nutrient Cycles and Food Webs. 1985. p. 372–377.

[91] Beszteri S., Thoms S., Benes V., Harms L., and Trimborn S., The Response of Three Southern Ocean Phytoplankton Species to Ocean Acidification and Light Availability: A Transcriptomic Study. Protist, 2018. 169(6): p. 958–975. doi: 10.1016/j.protis.2018.08.003 30453274

[92] Committee G.S., Guidelines for the Study of Climate Change Effects on HABs, in IOC Manuals and Guides. 2021, UNESCO-IOC/SCOR: Paris, France. p. 120.

