# Supplementary material for: Light-dependent variations in fatty acid profiles and gene expression in Antarctic microalgal cultures
Source: PLoS One. 2025 Jan 16;20(1):e0317044. doi: 10.1371/journal.pone.0317044 (PMC11737666; doi:10.1371/journal.pone.0317044)
Supplement: S1 File — (PDF) [file pone.0317044.s001.pdf]

Supporting Information

Light-Dependent Variations in Fatty Acid Profiles and Gene Expression in Antarctic Pennate Diatom and Dinoflagellate Microalgal Cultures

Jacqui Stuart[1, 2], Kirsty F. Smith[2], Matt Miller[2], John K. Pearman[2], Natalie Robinson[3], Lesley Rhodes[2], Lucy Thompson[2], Sarah Challenger[2], Nicole Parnel [4], Ken G. Ryan[1]

1) School of Biological Sciences, Victoria University of Wellington, Wellington, New Zealand. 2) Cawthron Institute, Nelson, New Zealand. 3) National Institute of Water and Atmospheric Research (NIWA), Wellington, New Zealand 4) Lincoln University, Lincoln, New Zealand.

S1 Fig. Maximum likelihood phylogenies. 2

S2 Table. list of all the light conditions. 3

S3 Table. Microalgae sample dry weights for Fatty acid (FA) analysis. 3

S4 Table. Percent contribution of fatty acids for isolates 4

S5 Fig. Principle component analysis (PCoA) of transcriptomes 5

S6 Fig. Differential analysis of KEGG pathways for transcriptomes. 5

S7 Fig. Differentially expressed genes for photosynthesis in *Nitzschia* cf. *biundulata*. 6

S8 Fig. Differentially expressed genes for photosynthesis in *Polarella glacialis*. 7

S9 Fig. Differentially expressed genes for FA biosynthesis in *N.* cf *biundulata*. 8

S10 Fig. Differentially expressed genes for FA elongation in *N. cf biundulata* 9

S11 Fig. Differentially expressed genes for FA degradation in *N. cf biundulata.* 10

S12 Fig. Differentially expressed genes for FA degradation in *P. glacialis*. 11

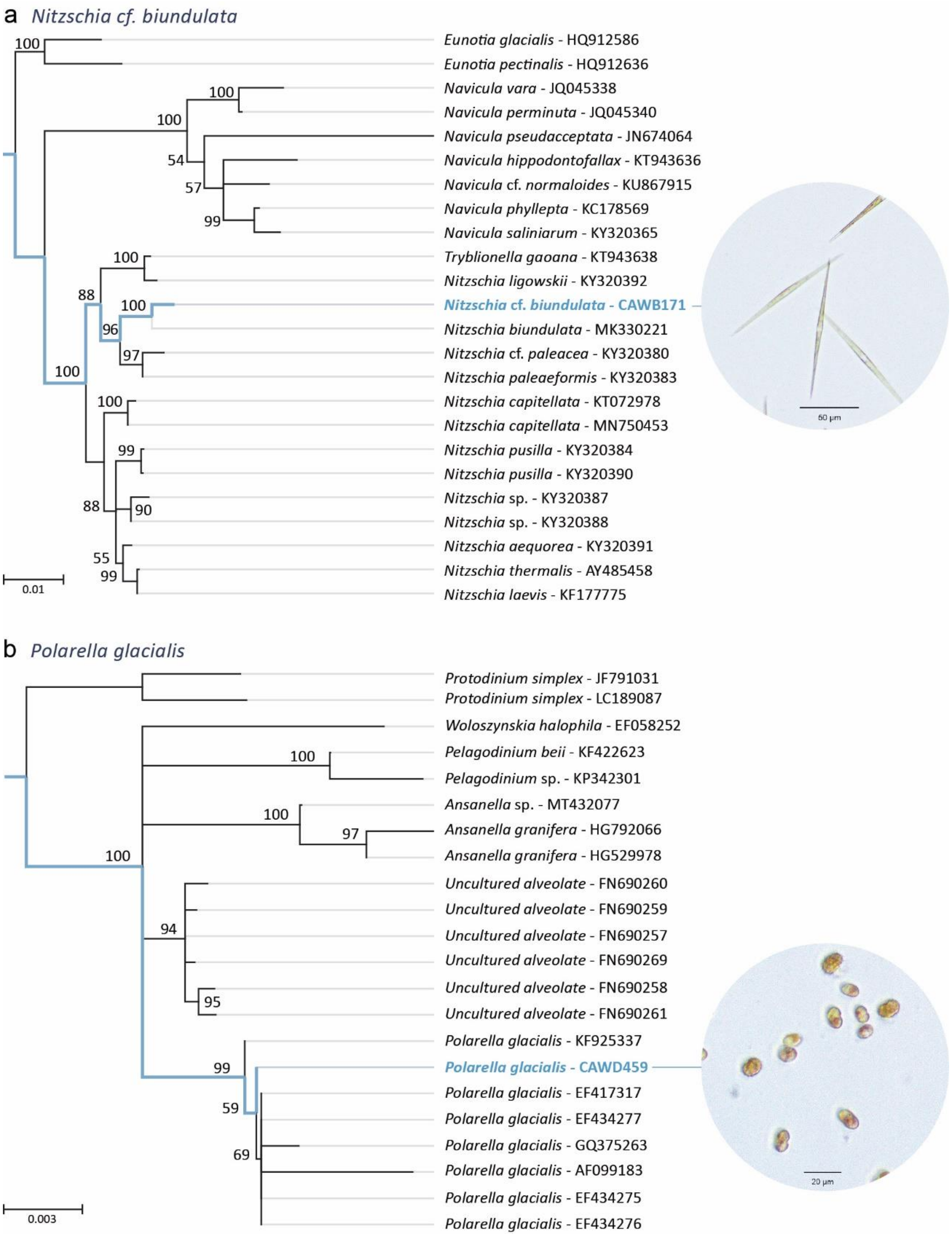

S1 Fig. Maximum likelihood phylogenies and light microscopy of a) *Polarella glacialis* (CICCM ID: CAWD459; GenBank Accession No: PP922274) and b) *Nitzschia* cf. *biundulata* (CICCM ID: CAWB171; GenBank Accession No: PP928079) used in the light levels experiment. Both isolates are maintained at the Cawthron Institute Culture Collection of Microalgae (http://cultures.cawthron.org.nz/ciccm/). Bootstrap support values are reported on the nodes. Analyses were conducted using 10,000 bootstrap replications. The scale indicates the number of substitutions per site.

S2 Table. list of all the light conditions for each isolate in this experiment, with all grown at 4 ± 1 °C in 400 mL of f2 media (35.3ppt).

Species	Light conditions	light (µmol)
Polarella glacialis	Low	1.5 ± 1
Polarella glacialis	Mid	10 ± 1
Polarella glacialis	High	90 ± 1
Nitzschia cf. biundulata	Low	1.5 ± 1
Nitzschia cf. biundulata	Mid	10 ± 1
Nitzschia cf. biundulata	High	90 ± 1

S3 Table. Microalgae sample dry weights for Fatty acid analysis.

Sample ID	Isolate	Species	treatment	rep.	Analysis Weight (mg)
B171 C1	CAWB171	*Nitzschia sp.*	High light	1	30.2
B171 C2	CAWB171	*Nitzschia sp.*	High light	2	25.6
B171 C3	CAWB171	*Nitzschia sp.*	High light	3	24.6
B171 C4 a	CAWB171	*Nitzschia sp.*	High light	4a	17.2
B171 C4 b	CAWB171	*Nitzschia sp.*	High light	4b	9.3
B171 C5	CAWB171	*Nitzschia sp.*	High light	5	20.9
B171 M1	CAWB171	*Nitzschia sp.*	Mid light	1	13.1
B171 M2	CAWB171	*Nitzschia sp.*	Mid light	2	21.0
B171 M3	CAWB171	*Nitzschia sp.*	Mid light	3	25.3
B171 M4	CAWB171	*Nitzschia sp.*	Mid light	4	20.9
B171 M5	CAWB171	*Nitzschia sp.*	Mid light	5	24.8
B171 L1	CAWB171	*Nitzschia sp.*	Low light	1	13.9
B171 L2	CAWB171	*Nitzschia sp.*	Low light	2	20.5
B171 L3	CAWB171	*Nitzschia sp.*	Low light	3	13.3
B171 L4	CAWB171	*Nitzschia sp.*	Low light	4	24.4
B171 L5	CAWB171	*Nitzschia sp.*	Low light	5	21.8
A33 C1	22-A33	*Polarella sp.*	High light	1	12.4
A33 C2	22-A33	*Polarella sp.*	High light	2	14.8
A33 C3	22-A33	*Polarella sp.*	High light	3	13.1
A33 C4	22-A33	*Polarella sp.*	High light	4	14.3
A33 C5	22-A33	*Polarella sp.*	High light	5	8.9
A33 M1	22-A33	*Polarella sp.*	Mid light	1	8.0
A33 M2	22-A33	*Polarella sp.*	Mid light	2	12.5
A33 M3	22-A33	*Polarella sp.*	Mid light	3	12.0
A33 M4	22-A33	*Polarella sp.*	Mid light	4	11.4
A33 M5	22-A33	*Polarella sp.*	Mid light	5	9.0
A33 L1	22-A33	*Polarella sp.*	Low light	1	7.2
A33 L2	22-A33	*Polarella sp.*	Low light	2	5.2
A33 L3	22-A33	*Polarella sp.*	Low light	3	6.2
A33 L4	22-A33	*Polarella sp.*	Low light	4	7.1
A33 L5	22-A33	*Polarella sp.*	Low light	5	3.0
QC	NA	*FAB QC #06*	NA	1	20.9
BL	NA	*BLANK*	Blank	1	0.0

S4 Table. Percent contribution of fatty acids for cultures of isolates a) *Nitzschia* cf. *biundulata* (CAWB171), and b) *Polarella glacialis* (CAWD) under High, mid and low light treatments.

Fatty Acids (%)	Nitzschia			Polarella		
	High	Mid	Low	High	Mid	Low
C12:0 Lauric	0.65 ± 0.05	1.64 ± 0.05	1.56 ± 0.09	0.30 ± 0.12	1.18 ± 0.13	0.78 ± 0.18
C14:0 Myristic	4.50 ± 0.24	5.14 ± 0.15	5.78 ± 0.37	11.58 ± 0.40	6.48 ± 0.57	7.08 ± 1.1
C16:0 Palmitic	16.7 ± 1.65	10.42 ± 0.31	9.08 ± 0.73	8.56 ± 0.69	6.48 ± 0.29	8.22 ± 0.72
C16:1 Palmitoleic	35.1 ± 1.33	25.74 ± 1.57	28.32 ± 1.68	0.4 ± 0.1	0.32 ± 0.04	0.84 ± 0.18
C16:2n-4 Hexadecadienoic	3.60 ± 0.19	5.00 ± 0.14	3.74 ± 2.08	0 ± 0	0 ± 0	0 ± 0
C18:0 Stearic	0.37 ± 0.08	0.36 ± 0.13	0.58 ± 0.19	1.52 ± 0.18	0.92 ± 0.18	0.84 ± 0.4
C18:1n-9C Oleic	3.00 ± 0.13	7.72 ± 0.39	5.16 ± 1.05	8.68 ± 1.08	5.48 ± 1.08	12.62 ± 2.64
C18:2n-6C Linoleic	2.53 ± 3.92	0 ± 0	0 ± 0	0 ± 0	0 ± 0	1.1 ± 2.46
C18:4n-3 Stearidonic	1.05 ± 0.08	0.54 ± 0.05	0.28 ± 0.08	2.98 ± 0.22	1.28 ± 0.08	1.54 ± 0.21
C20:3n-6 Eicosatrienoic	0.17 ± 0.10	0.2 ± 0.0	0.08 ± 0.04	n.d	n.d	n.d
C18:5n-3 Octadecapentaenoic	n.d	n.d	n.d	24.58 ± 1.71	44.24 ± 3.37	28.2 ± 5.07
C20:4n-6 Arachidonic	0.98 ± 0.20	1.36 ± 0.18	0 ± 0	0 ± 0	0 ± 0	0 ± 0
C20:5n-3 Eicosapentaenoic	17.57 ± 2.0	18.6 ± 0.85	19.1 ± 2.69	3.1 ± 0.24	4.44 ± 0.23	4 ± 0.2
C22:6N3 Docosahexaenoic	1.33 ± 0.33	1.88 ± 0.13	2.22 ± 0.36	22.16 ± 0.85	19.68 ± 1.03	21.78 ± 3.87
Σ SFA	22.93 ± 1.87	18.42 ± 0.68	18.68 ± 1.39	22.26 ± 0.83	15.5 ± 0.88	17.66 ± 1.24
Σ MUFA	38.52 ± 1.35	34.00 ± 1.20	34.34 ± 0.80	9.96 ± 1.26	6.94 ± 1.06	14.34 ± 2.53
Σ PUFA	28.28 ± 4.97	28.32 ± 1.09	26.22 ± 4.89	56.56 ± 2.61	70.94 ± 2.43	58.12 ± 1.83
Σ Omega 3s	20.5 ± 2.53	21.32 ± 0.93	21.62 ± 2.91	28.42 ± 0.92	25.54 ± 0.98	27.64 ± 3.99
Σ FA identified	89.73 ± 3.61	80.74 ± 1.34	79.26 ± 4.29	88.92 ± 1.19	93.66 ± 0.74	90.8 ± 3.38

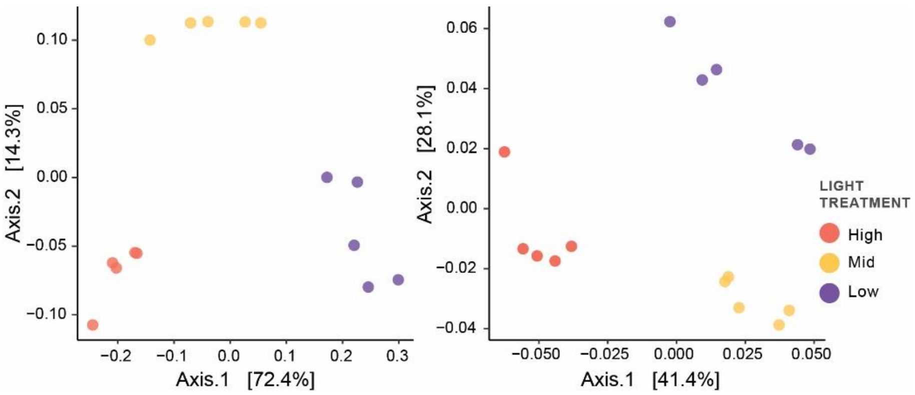

S5 Fig. Principal component analysis (PCoA) of transcriptomes from high, mid and low light experiment conditions for a) *Nitzschia* cf. *biundulata* and b) *Polarella glacialis*

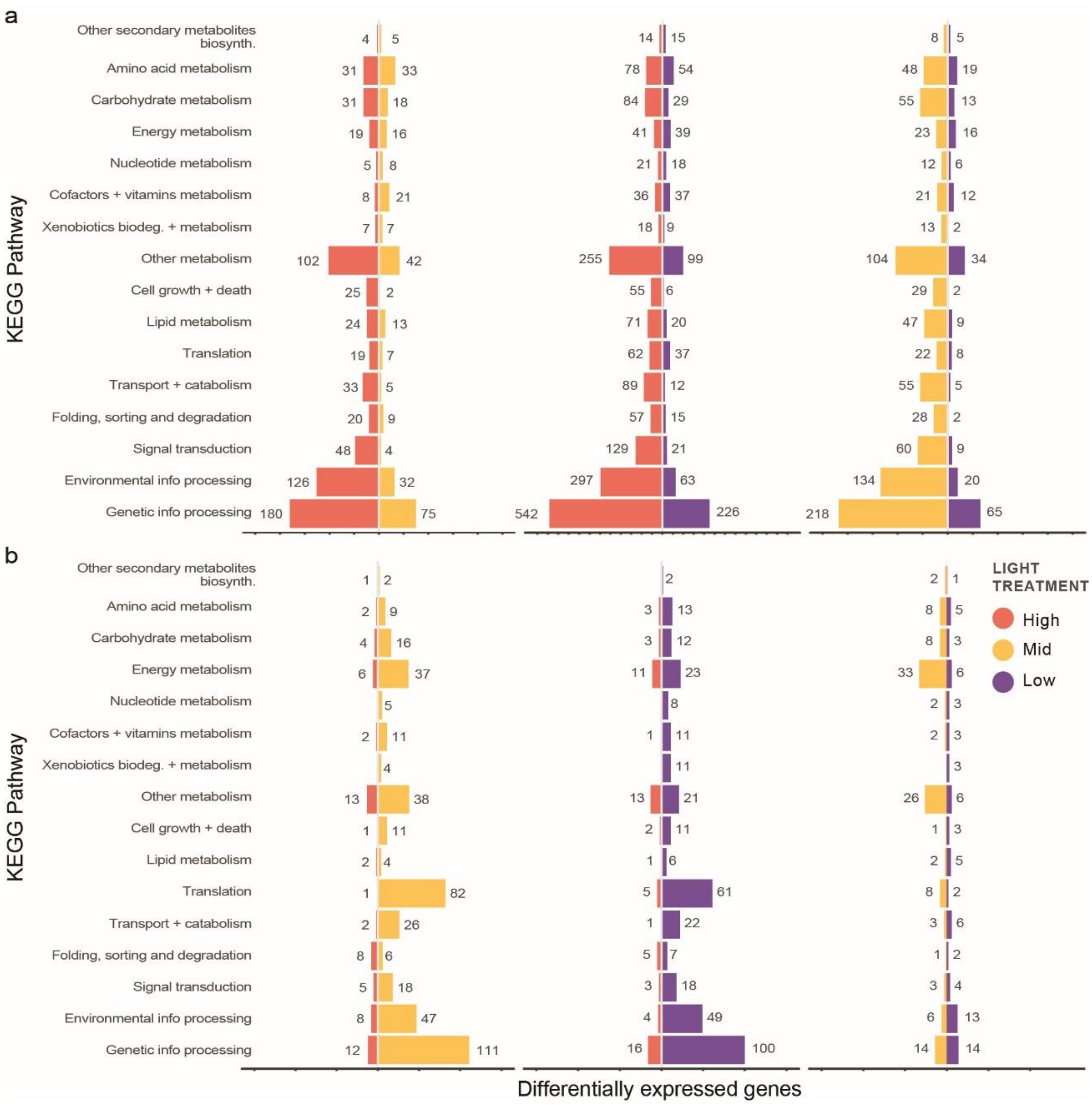

S6 Fig. Differential analysis of KEGG pathways for transcriptomes of a) *Nitzschia* cf. *biundulata* and b) *Polarella glacialis* from high, mid, and low light experiment conditions.

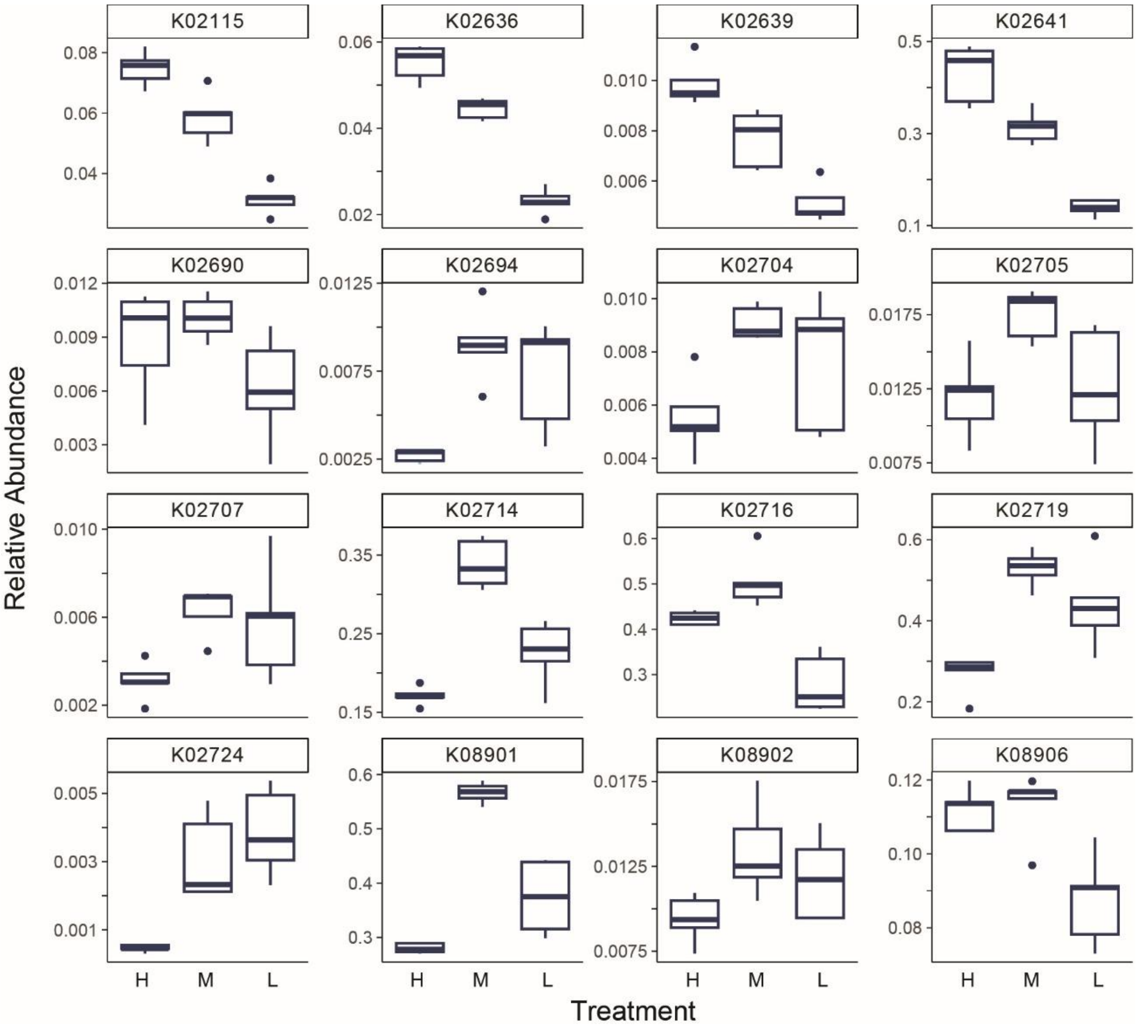

S7 Fig. Differentially expressed genes for photosynthesis in *Nitzschia* cf. *biundulata* cultures grown under high (H), mid (M) and low (L) light conditions.

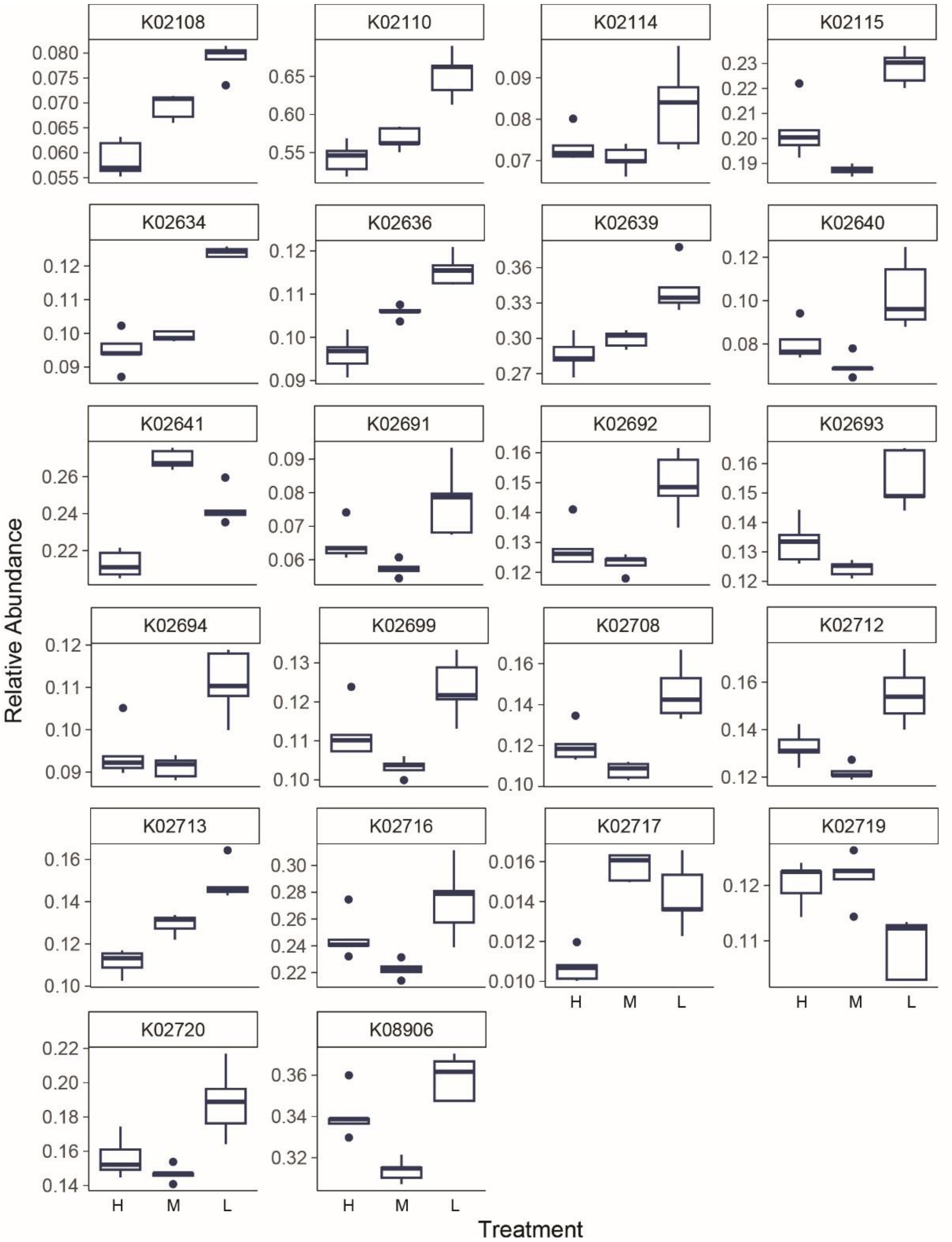

S8 Fig. Differentially expressed genes for photosynthesis in *Polarella glacialis* cultures grown under high (H), mid (M) and low (L) light conditions.

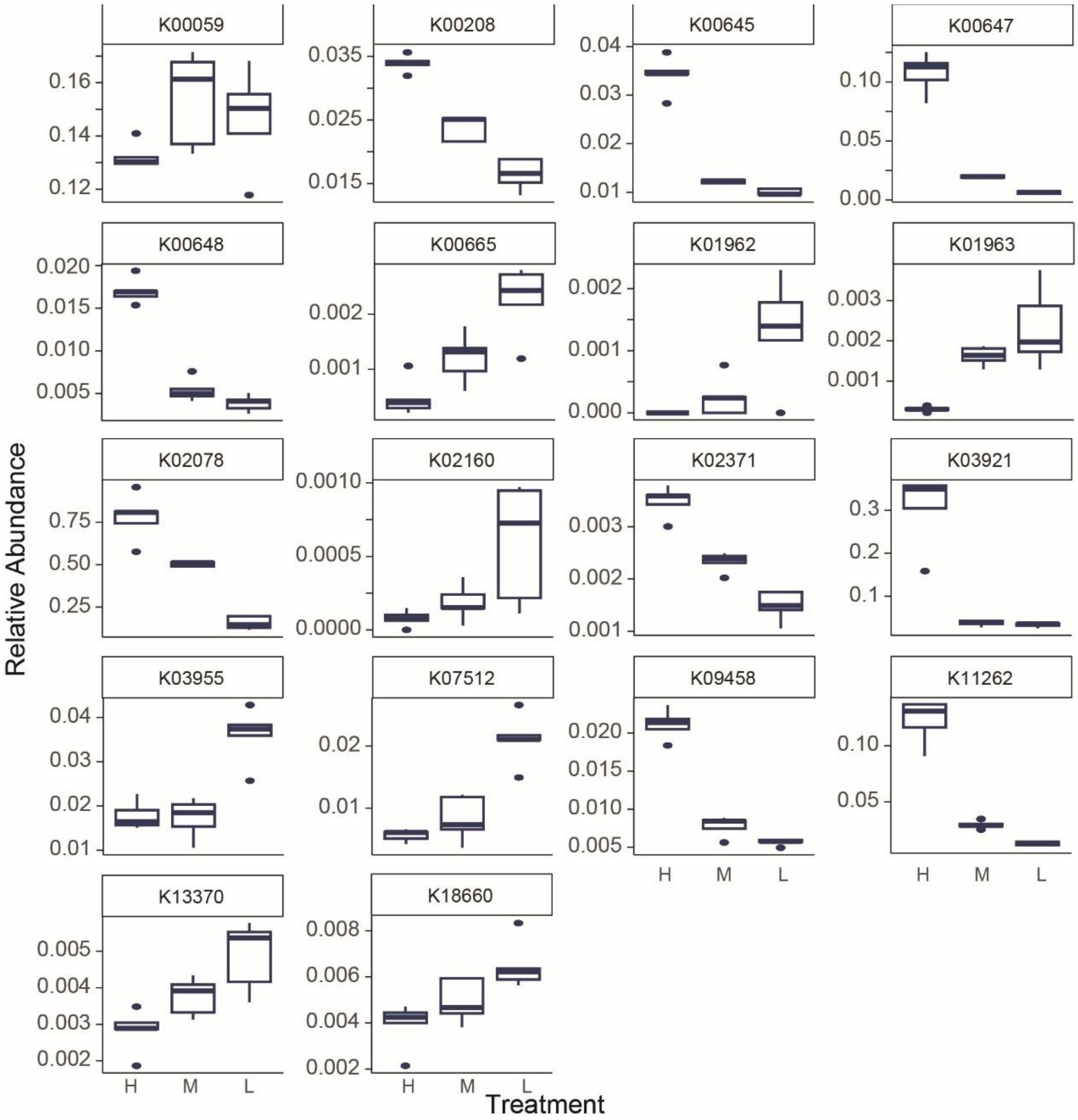

S9 Fig. Differentially expressed genes for fatty acid biosynthesis in *Nitzschia* cf. *biundulata* cultures grown under high (H), mid (M) and low (L) light conditions.

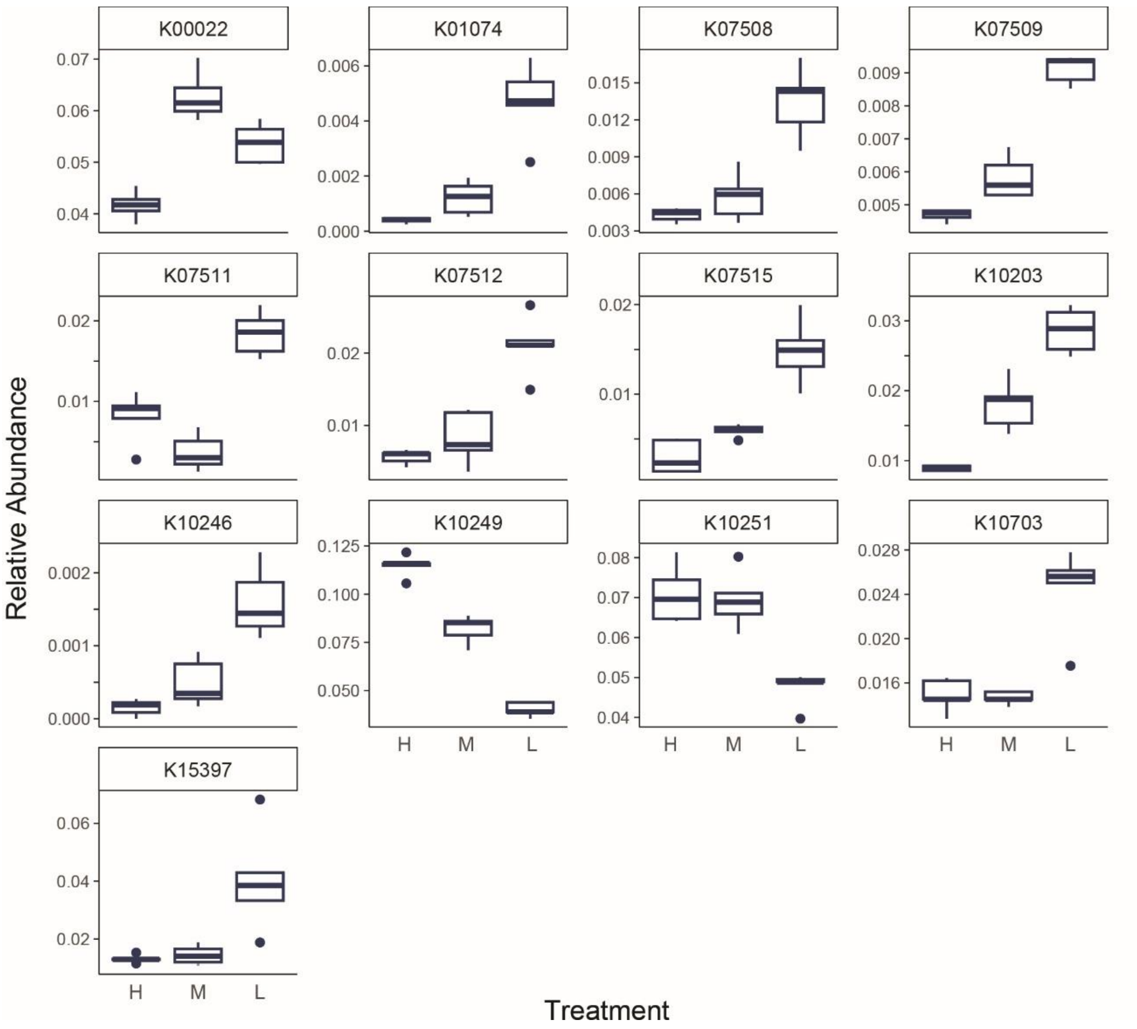

S10 Fig. Differentially expressed genes for fatty acid elongation in *Nitzschia* cf. *biundulata* cultures grown under high (H), mid (M) and low (L) light conditions.

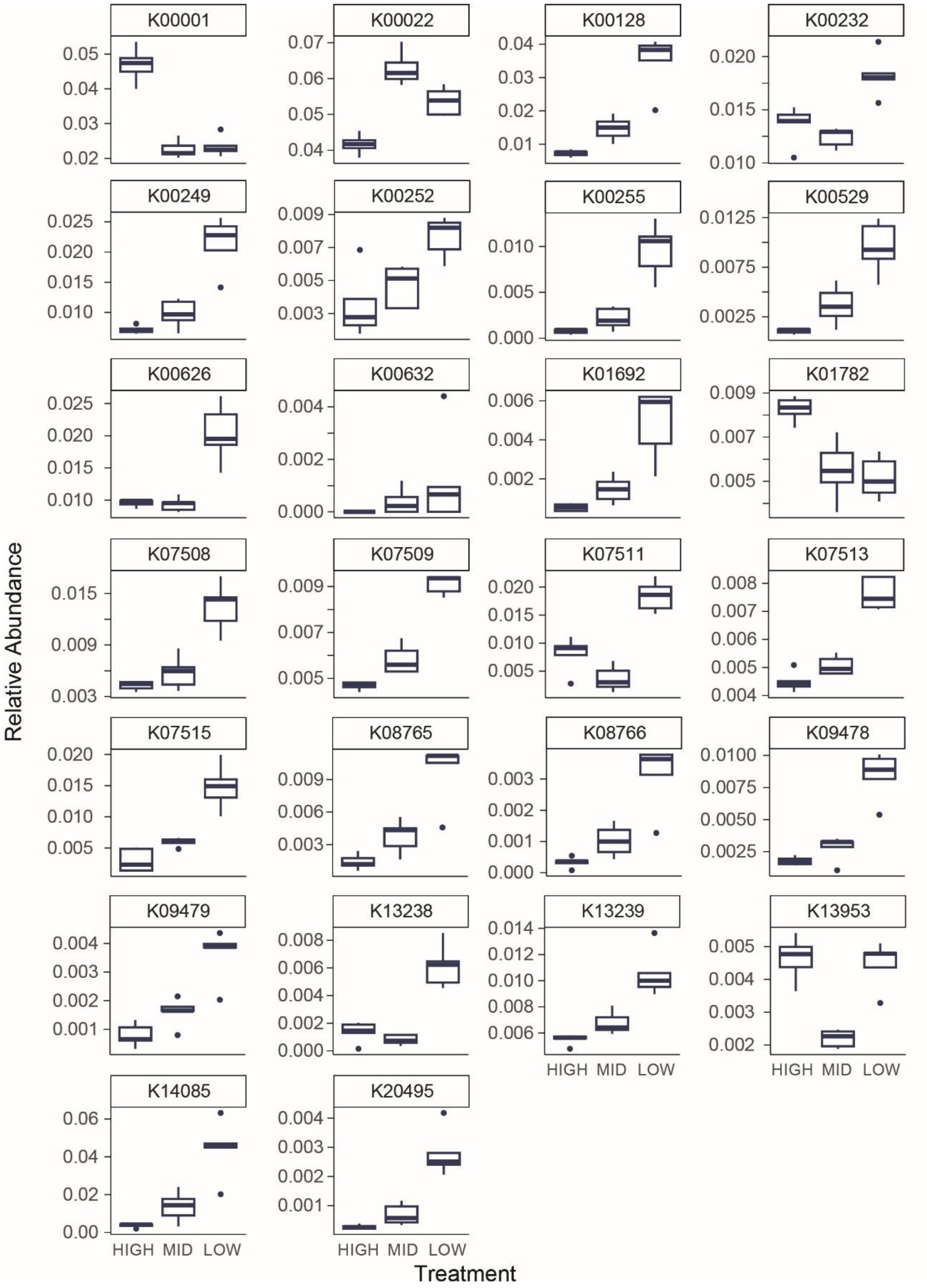

S11 Fig. Differentially expressed genes for fatty acid degradation in *Nitzschia* cf. *biundulata* cultures grown under high (H), mid (M) and low (L) light conditions.

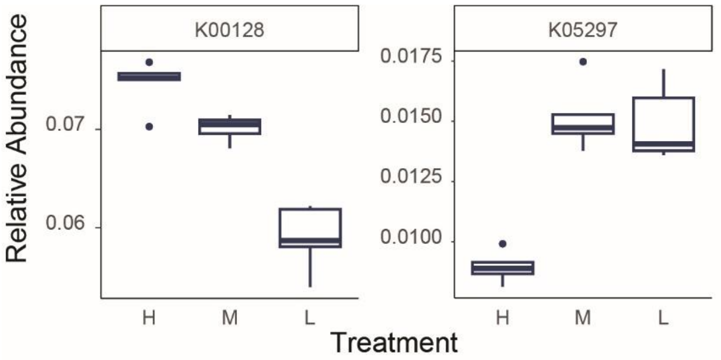

S12 Fig. Differentially expressed genes for fatty acid degradation in *Polarella glacialis* cultures grown under high (H), mid (M) and low (L) light conditions.
