# Supplementary figures and images for: Light-dependent variations in fatty acid profiles and gene expression in Antarctic microalgal cultures
Source: PLoS One. 2025 Jan 16;20(1):e0317044. doi: 10.1371/journal.pone.0317044 (PMC11737666; doi:10.1371/journal.pone.0317044)

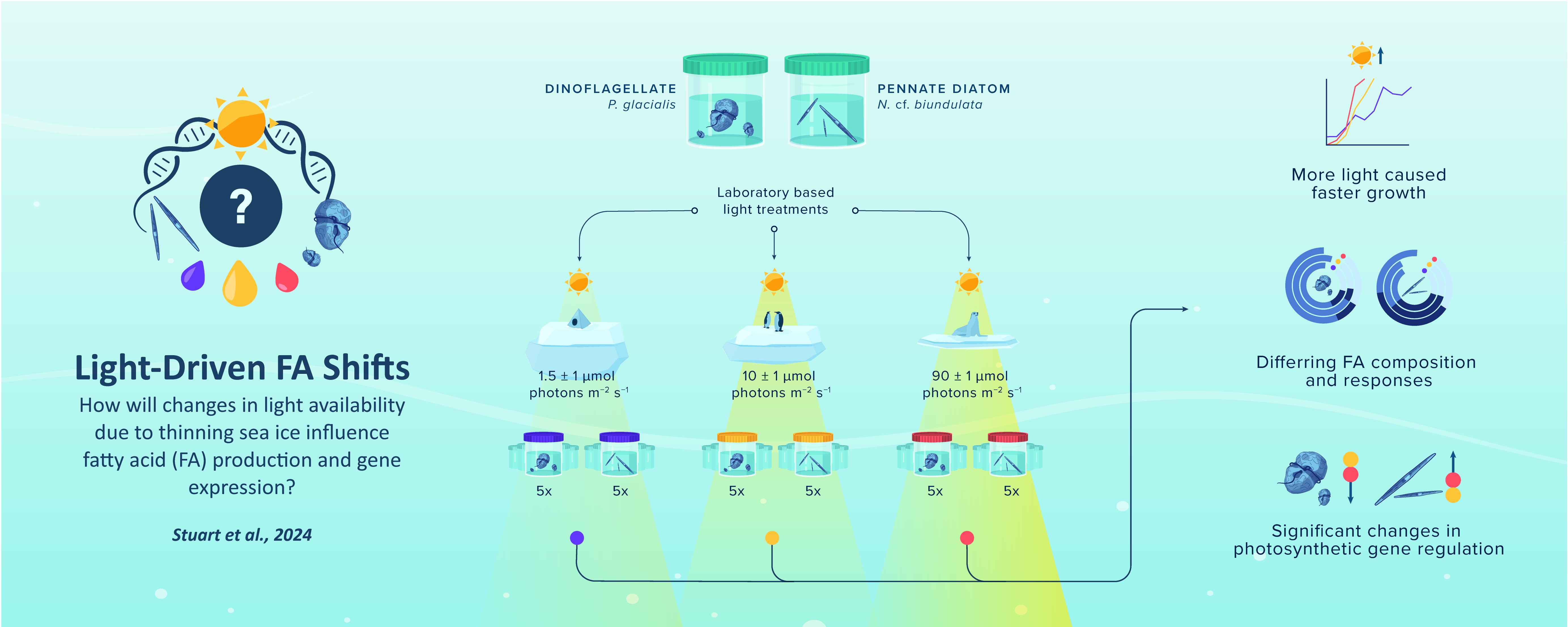

Supplement: S1 Graphical abstract — (TIF) [file pone.0317044.s002.tif]
